# Comprehensive genomic analysis of the *DUF4228* gene family in land plants and expression profiling of *ATDUF4228* under abiotic stresses

**DOI:** 10.1186/s12864-019-6389-3

**Published:** 2020-01-03

**Authors:** Qi Yang, Xiaocui Niu, Xiaona Tian, Xiujuan Zhang, Jingyu Cong, Ruigang Wang, Guosheng Zhang, Guojing Li

**Affiliations:** 1 College of Life Sciences, Inner Mongolia Key Laboratory of Plant Stress Physiology and Molecular Biology, Inner Mongolia Agricultural University, Hohhot, China; 2 Forestry College, Inner Mongolia Agricultural University, Hohhot, China

**Keywords:** DUF4228, Phylogenetic analysis, Genomic analysis, Abiotic stress

## Abstract

**Background:**

Domain of unknown function (DUF) proteins represent a number of gene families that encode functionally uncharacterized proteins in eukaryotes. The *DUF4228* gene family is one of these families in plants that has not been described previously.

**Results:**

In this study, we performed an extensive comparative analysis of DUF4228 proteins and determined their phylogeny in the plant lineage. A total of 489 high-confidence DUF4228 family members were identified from 14 land plant species, which sub-divided into three distinct phylogenetic groups: group I, group II and group III. A highly conserved DUF4228 domain and motif distribution existed in each group, implying their functional conservation.

To reveal the possible biological functions of these *DUF4228* genes, 25 *ATDUF4228* sequences from *Arabidopsis thaliana* were selected for further analysis of characteristics such as their chromosomal position, gene duplications and gene structures. Ka/Ks analysis identified seven segmental duplication events, while no tandemly duplication gene pairs were found in *A. thaliana*. Some cis-elements responding to abiotic stress and phytohormones were identified in the upstream sequences of the *ATDUF4228* genes. Expression profiling of the *ATDUF4228* genes under abiotic stresses (mainly osmotic, salt and cold) and protein-protein interaction prediction suggested that some *ATDUF4228* genes are may be involved in the pathways of plant resistance to abiotic stresses.

**Conclusion:**

These results expand our knowledge of the evolution of the *DUF4228* gene family in plants and will contribute to the elucidation of the biological functions of *DUF4228* genes in the future.

## Background

Domains of unknown function (DUFs) are a large set of families within the Pfam database that do not include any proteins of known function [1]. In 1998, Chris Ponting first named DUF1 and DUF2 and added them to the SMART database. Subsequently, the functions of DUF1 and DUF2 were identified, and they have since been renamed the GGDEF domain and EAL domain, respectively [1, 2]. In recent years, the number of DUF families has increased rapidly due to the large number of species genomes were sequenced. The Pfam database (version 32.0) now includes 17,929 families, almost 22% (3961 out of 16,712) of which are populated by DUF families [3].

A DUF family will be renamed when the function of at least one of its members has been experimentally determined, but the number of newly added DUFs is much greater than that of renamed DUFs due to the development of sequencing technology [4]. The DUF families have been collected together in the Pfam database using the prefix DUF followed by a number, such as DUF1218 [5] or DUF1313 [6]. Some DUF families are widely distributed, and some are only found in bacteria or eukaryotes [7].

Comprehensive genomic analysis enables researchers to understand the origin, evolution, and biological functions of a gene family. Although there have been numerous reports on other gene families in plants, there have been few reports of comprehensive genomic analyses of DUF families. Such analyses have been reported for *DUF221*, *DUF810*, *DUF866*, *DUF936* and *DUF1618* from *Oryza sativa* [8–12], the *DUF481* and *DUF724* gene families from *Arabidopsis thaliana* [13, 14], and *DUF1313* genes from 81 photoautotrophic species [6].

DUFs have a variety of functions in plants, and recent studies have shown that some DUFs play important roles in plant abiotic stress responses. In *A. thaliana*, Xin’s results indicate that *ESK1* (*AT3g55990*), which belongs to the *DUF231* gene family, is a novel negative regulator of cold acclimation [15]. Another study in *A. thaliana* showed that ABA-mediated drought stress was reduced after suppressing the expression of *ATRDUF1* and *ATRDUF2* (both are RING-DUF1117 E3 ubiquitin ligases) [16]. *OsSIDP366*, a *DUF1644* gene from rice, positively regulates responses to drought and salt stresses, with transgenic rice plants overexpressing *OsSIDP366* showing enhanced drought and salinity tolerance [17]. *OsSGL* confers enhanced drought tolerance in transgenic *O. sativa* and *A. thaliana*, and several stress-responsive genes were shown to be significantly altered in transgenic rice [18]. Other *DUF* genes in *O. sativa* have also been shown to be associated with abiotic stress, such as *SIDP361* (*DUF1644*) [19], *OsDSR2* (*DUF966*) [20] and *OsDUF810.7* [10]. In *Medicago truncatula*, three members of the ST family (DUF2275) are ubiquitous during development and modulated by nutritional status (*MtST1*) and dehydration (*MtST2* and *MtST3*) [21]. Over-expression of *TaSRHP*, a salt-induced gene containing a DUF581 domain, in wild-type *A. thaliana* resulted in enhanced resistance to both salt and drought stresses [22]. The functions of DUFs in resisting abiotic stress have been reported not only in model plants but also in other plants species. For example, mutation of *DUF1517* in *A. thaliana* resulted in enhanced sensitivity to cold stress, while heterologous expression of *AmDUF1517* in *atduf1517* mutants significantly rescued their cold-sensitive phenotypes [23]. *LcFIN1*, a DUF761 encoding gene from sheep grass, was shown to be directly regulated by LcCBF1 and to enhance plant adaptation to cold stress [24].

DUF4228 domain-containing proteins are exclusively present in plant genomes. As of March 2019, a total of 2882 genes belonging to the DUF4228 family (Pfam accession: 14009) could be retrieved from 80 species in the Pfam database. To date, only one *DUF4228* gene from plants has been reported*. MsDUF*, a novel stress-responsive gene belonging to the *DUF4228* gene family from *Medicago sativa*, may act as a negative regulator in the control of seed vigour and responses to osmotic stress in plants [25].

Thus far, there has been no systematic study on the evolution, classification of function of this family. In this study, we performed a comprehensive analysis of the *DUF4228* gene family in 14 representative land plant species, including phylogenetic and conserved motif analysis. Furthermore, the chromosomal distribution, gene duplication, cis-elements in promoters and expression profiles under abiotic stresses were analysed for 25 *DUF4228* genes from *A. thaliana* to reveal their possible biological functions.

The results obtained here will broaden our understanding of the roles of *DUF4228* genes and provide a framework for further functional investigation of these genes under abiotic stress in plants.

## Result

### Identification of the *DUF4228* gene family from plants

To investigate the copy number variation of the *DUF4228* genes during plant evolution, a comprehensive search was conducted for *DUF4228* genes across plant lineages, including 16 representative species from Chlorophyta, Bryophyta, lycophytes, Gymnospermae and angiosperms (basal angiosperms, eudicots and monocots) (Additional file 1: Table S1).

**Fig. 1:**
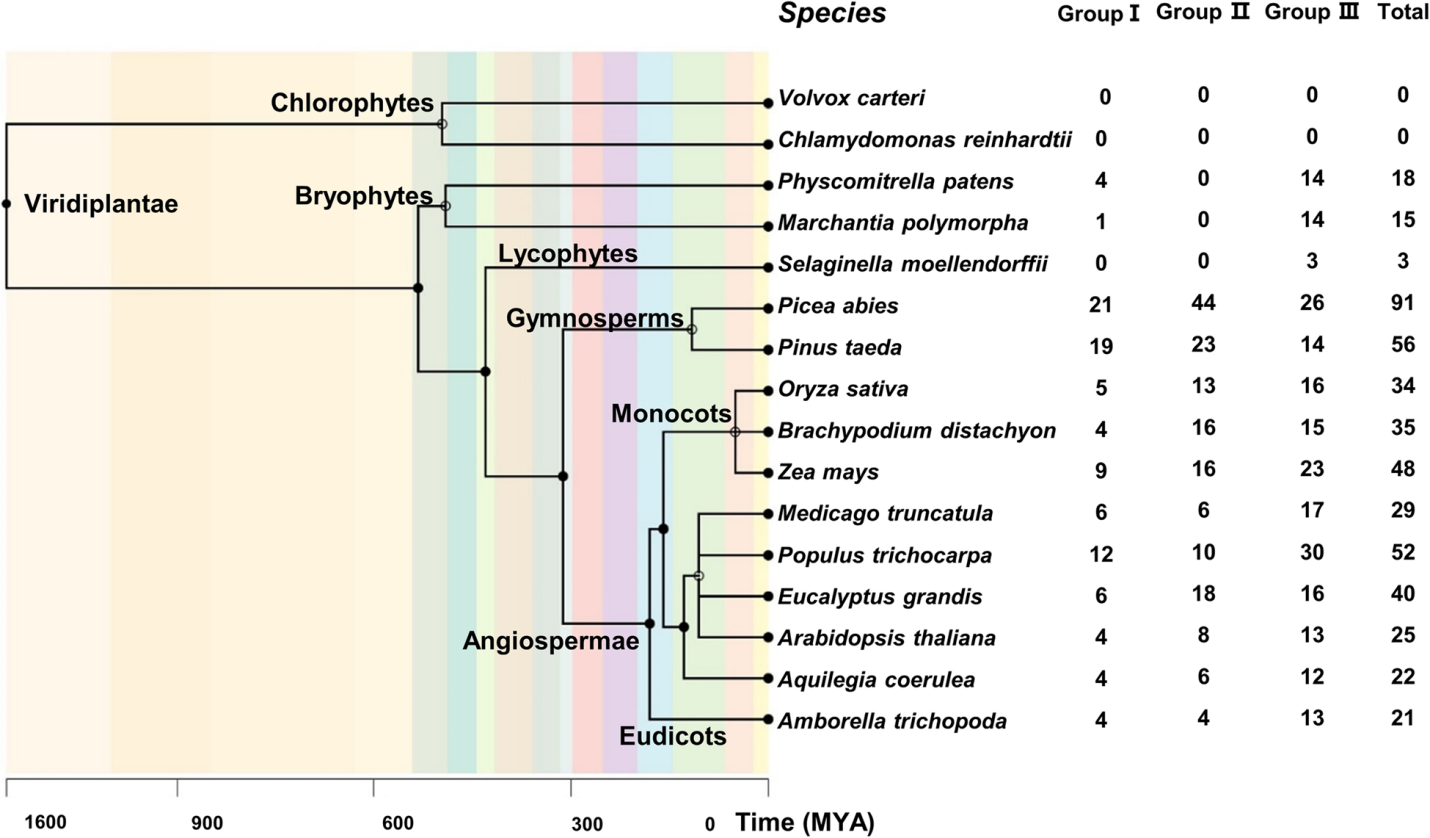
The phylogeny of the 16 plants analysed in this study and the number of *DUF4228* genes identified. The order of tree branches and divergence time are derived from the TimeTree database (http://timetree.org/)

HMM (Hidden Markov Model) searches were used to identify candidate *DUF4228* genes from plants. All of the candidates and their encoded protein features are listed in Table S2 (Additional file 2: Table S2). Then, the candidate genes with low visibility and incomplete domains were removed. For example, in *A. thaliana* and *O. sativa*, 28 and 39 candidate *DUF4228* genes were initially identified from the genomes through HMM searches, and three and five genes, respectively, were excluded due to low domain coverage. Finally, 25 and 34 *DUF4228* genes with high confidence were retained from these species (Additional file 2: Table S2). In this study, no *DUF4228* genes were identified from two Chlorophyta, *Volvox carteri* and *Chlamydomonas reinhardtii*, but a total of 489 high-confidence full-length *DUF4228* genes were identified from 14 land plant species, including the four major land plant lineages, of the bryophytes, lycophytes, gymnosperms and angiosperms (Fig. 1 and Additional file 2: Table S2). These *DUF4228* genes with high confidence were used for the evolutionary analysis.

As shown in Fig. 1, the two less-advanced groups of early land plants, mosses and lycophytes, contained fewer *DUF4228* genes than the other land plants included in this study. Among these groups, the lycophyte *Selaginella moellendorffii* harboured only three *DUF4228* genes, ranking last among 14 land plant species. In contrast, two gymnosperms, *Picea abies* and *Pinus taeda*, presented the most members of the *DUF4228* gene family, with 91 and 56 respectively. Among the nine angiosperms, the number of *DUF4228* genes ranged from 21 (*Amborella trichopoda*) to 52 (*Populus trichocarpa*), which was greater than the numbers in bryophytes and the pteridophytes lower than that in gymnosperms. Overall, the number of *DUF4228* genes increased significantly from bryophytes to gymnosperms and angiosperms.

### Phylogenetic analysis and classification of the *DUF4228* gene family

**Fig. 2:**
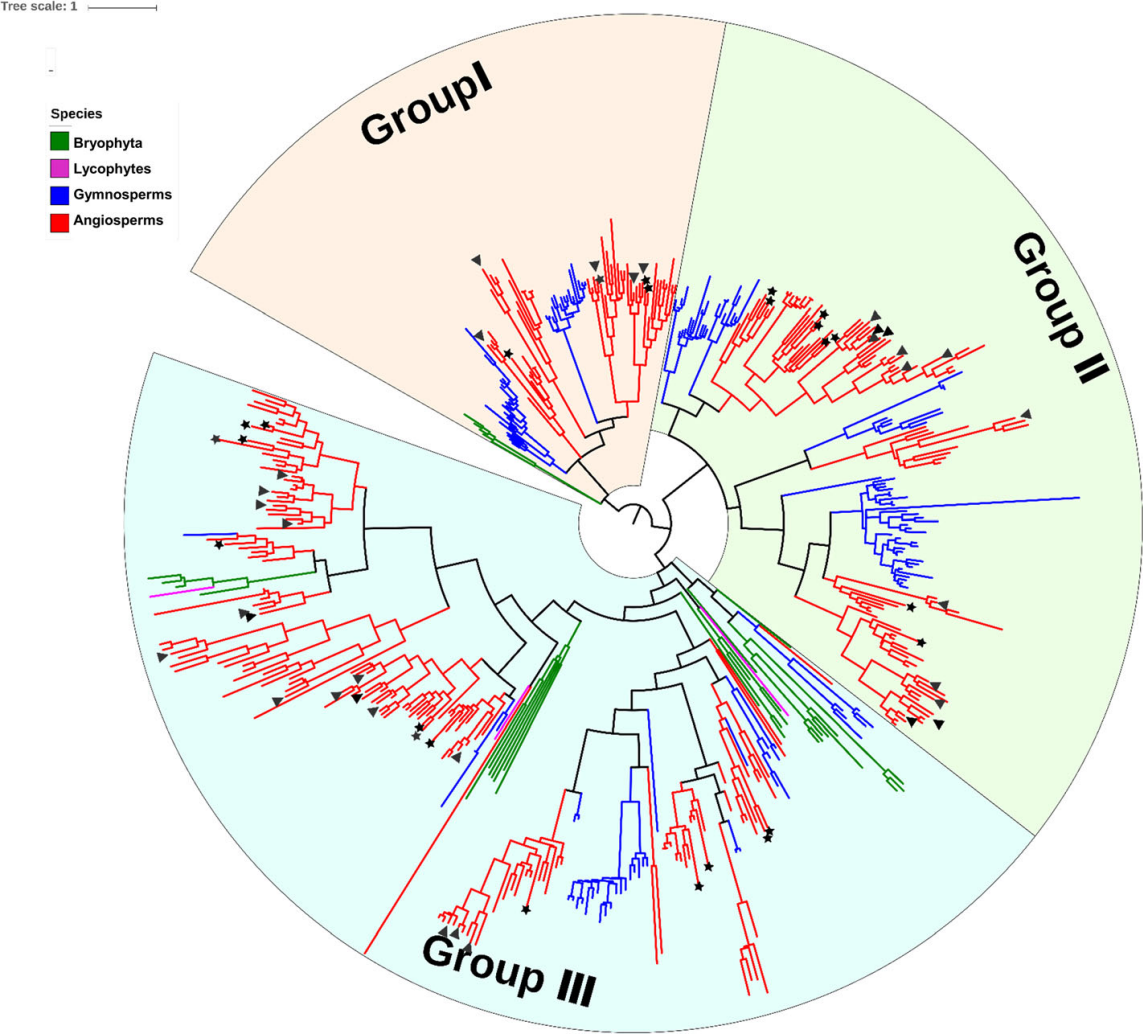
Phylogenetic classification of *DUF4228* genes in land plant lineages. A phylogenetic tree was constructed using the ML method with IQ-tree. Green lines represent bryophytes, pink lines represent pteridophytes, blue lines represent pteridophytes and red lines represent angiosperms. Twenty-five and 34 *DUF4228* genes in *A. thaliana* and *O. sativa* were labelled with black stars and triangles

To reveal the evolutionary relationships of the *DUF4228* genes among the 14 land plant lineages used in this study, we constructed a phylogenetic tree via the maximum likelihood method. The tree showed that these DUF4228 members can be divided into three groups, group I, group II and group III (Fig. 2 and Additional file 3: Figure S1). The number of *DUF4228* genes in each plant species is listed in Fig. 1. For example, in *A. thaliana*, there are 4, 8, and 13 genes in group I, group II and group III, respectively. In *O. sativa*, there are 5, 13 and 16 genes in these groups. In Fig. 2, 25 and 34 *DUF4228* genes from *A. thaliana* and *O. sativa* are labelled with black stars and triangles, respectively.

As shown in Fig. 1 and Fig. 2, in each of the plant species included in this study, the number of *DUF4228* genes belonging to group I was lowest among the three groups, except in *Pinus taeda*. Therefore, among all 489 genes, the number of *DUF4228* genes belonging to group I (99/489) was significantly lower than the number in either group II (164/489) or group III (226/489).

The three *DUF4228* genes from the lycophyte *S. moellendorffii* all belonged to in group III, while the *DUF4228* genes from *Marchantia polymorpha* and *Physcomitrella patens* belonged to both group I and group III. In particular, the genes in group II came only from angiosperms and gymnosperms, indicating that the *DUF4228* genes belonging to group II are unique to Spermatophyta.

Most of the *DUF4228* genes identified from the same plant lineage were clustered together (Fig. 2 and Additional file 3). For example, the *DUF4228* genes from angiosperms were clustered together and clearly separated from other plant lineages. The *DUF4228* genes from gymnosperms (blue lines) were clearly clustered on a large branch together with those from angiosperms, especially for group I and group II, because both groups to Spermatophyta. Similarly, among the angiosperms, the *DUF4228* genes from monocots and those from dicots were distinctly clustered on different branches. These results indicate that the evolutionary analysis of the *DUF4228* genes is in good agreement with the plant classification.

### The DUF4228 domain and conserved motifs in DUF4228 family members

Detailed information on the domains of the 489 DUF4228 protein sequences was obtained using the HMMER website and the distribution of the DUF4228 domain was plotted (Additional file 4: Figure S2). Among all 489 protein sequences used in this study, only the DUF4228 domain was present. Most DUF4228 proteins presented just one DUF4228 domain, but a few DUF4228 proteins from gymnosperms (*Picea abies* and *Pinus taeda*) presented 2 or 3 DUF4228 domains, such as PITA_000033628-RA, PITA_000033731-RA, PITA_000027867-RA and MA_10427211g0020. Among those proteins with only one DUF4228 domain, the DUF4228 domain occupied almost the total length of the sequence of each protein.

**Fig. 3:**
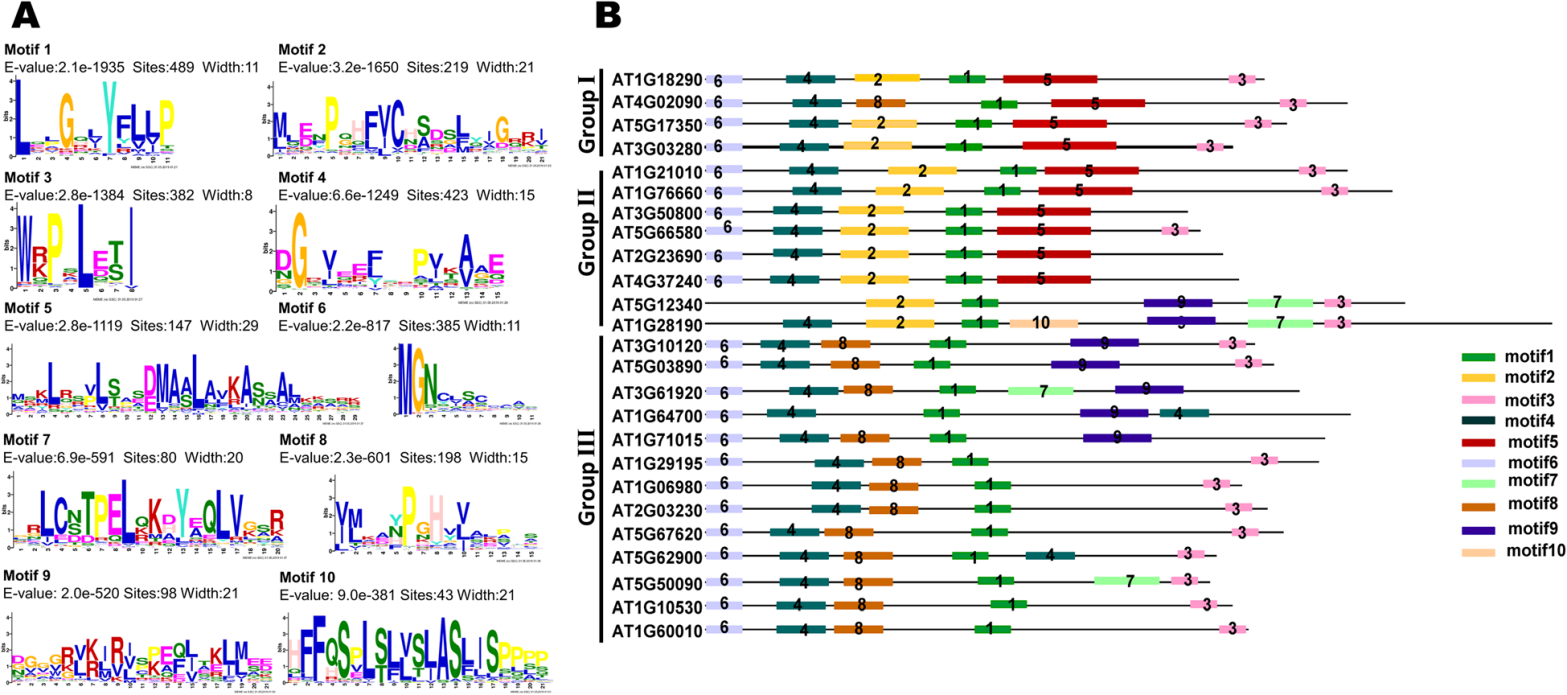
Sequence logos for the conserved motifs of DUF4228 proteins in plants (**a**) and the distribution of the conserved motifs in ATDUF4228 (**b**)

To reveal the correlation between sequence variation and evolutionary relationships, the 489 DUF4228 proteins from the 14 land plants were compared to identify the conserved motifs. The analysis of motifs which are representing features such as DNA-binding sites and protein interaction domains can help us to understand the common features of gene family sequences, and identify any novel conserved motif composition that might not be recorded in public databases [26, 27]. A total of 10 distinct conserved motifs were found, and the sequence logos for the conserved motifs and their distribution in each protein are illustrated in Fig. 3 and Additional file 5. Overall, the number of motifs distributed in group III was lower than those in the other two groups. Among the 10 conserved motifs, motif 1 was present in all 489 DUF proteins, while motif 3, motif 4 and motif 6 were present in most DUF4228 proteins. The distribution of the other motifs in the different groups shows a certain degree of specificity. For example, motif 2, motif 5 and motif 7 were mainly present in group I and group II, while motif 8 was mainly present in group III, and motif 10 was mainly present in group II. Most DUF4228 members belonged to the same group (especially the most closely related members) and shared common motif compositions, indicating potential functional similarities among these DUF4228 proteins. The distribution of the conserved motifs in the ATDUF4228 showed the same motif pattern as all 489 DUF4228 sequences. These specific motifs may also contribute to the functional divergence of *DUF4228* genes.

### Chromosomal position, synteny analysis and gene structure of *ATDUF4228* genes in *A. thaliana*

In *A. thaliana*, the *DUF4228* genes were widely distributed throughout the genome, but their distribution was uneven among chromosomes (Additional file 6: Figure S4). There were 10 *ATDUF4228* genes (40%) located on Chr (chromosome) 1, which was the maximum number found among all five chromosomes, while only 2 genes (8%) were located both on Chr 2 and Chr 4. Among the other *ATDUF4228* genes, 4 (16%) and 7 (28%) genes were located on chromosomes 3 and 5, respectively.

**Fig. 4:**
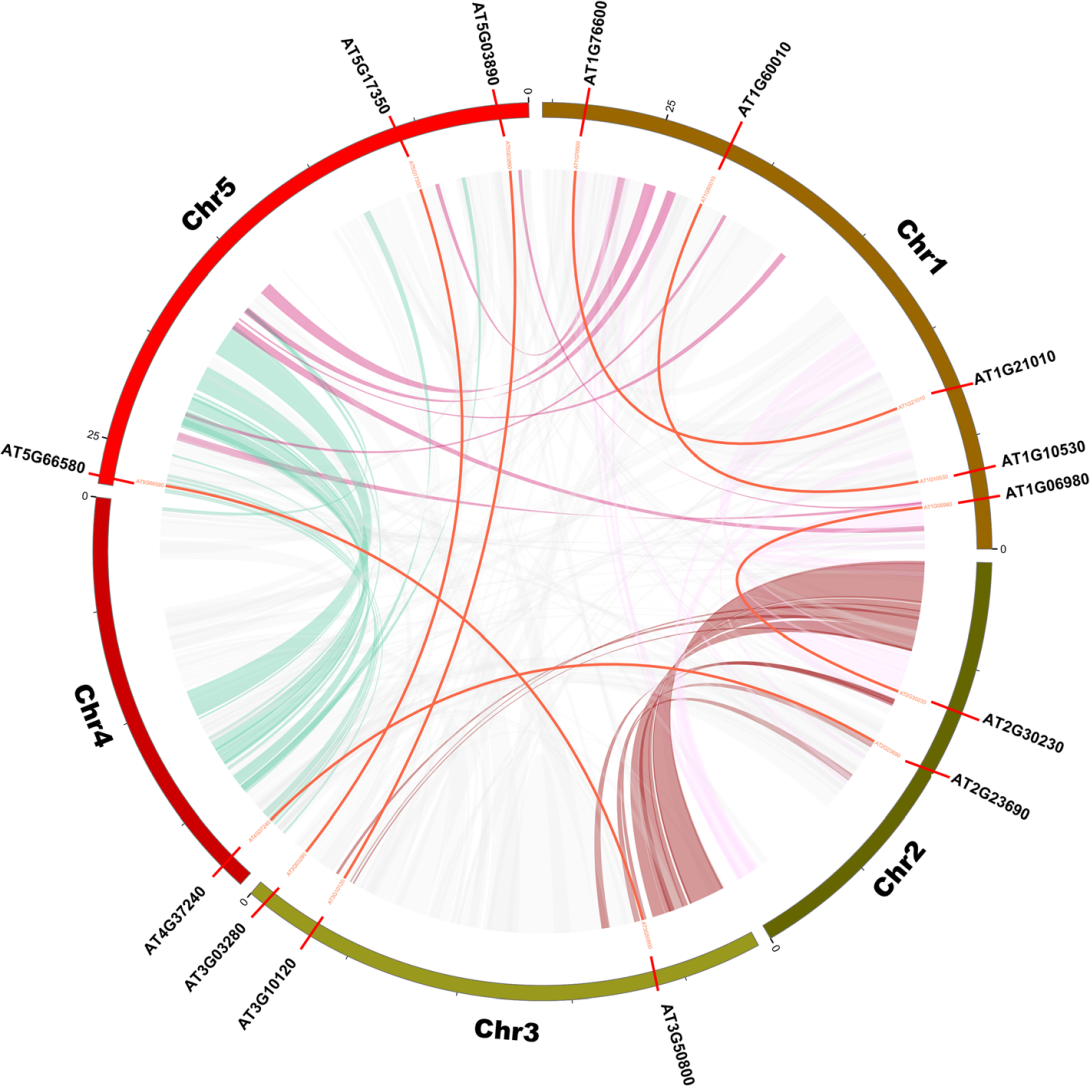
Mapping of *ATDUF4228* genes and the duplications among them on the *A. thaliana* chromosomes. The chromosomes of *A. thaliana* have been arranged in a circle, and duplications are represented by lines

In addition, duplicate gene pairs were searched for syntenic relationships using MCScanX [28]. As shown in Fig. 4, seven segmental duplications events involving 14 *ATDUF4228* genes (*AT1G06980*/*AT2G30230*, *AT1G10530*/*AT1G60010*, *AT1G21010*/*AT1G76660*, *AT2G23690*/*AT4G37240*, *AT3G03280*/*AT5G17350*, *AT3G10120*/*AT5G03890* and *AT3G50800*/*AT5G66580*) were identified, but no tandemly duplicated gene pairs were found. These results indicated that some *ATDUF4228* genes may have been generated by gene duplication and that segmental duplication events represent a major driving force of *ATDUF4228* evolution. The non-synonymous (Ka) and synonymous (Ks) substitution values and Ka/Ks ratios were calculated for the 7 identified duplicated gene pairs and are listed in Table 1. If the value of Ka/Ks equals 1, it indicates that genes are undergoing neutral selection; if the value of Ka/Ks is greater than 1, it means that genes are positively selected; if the value of Ka/Ks is less than 1, it shows that genes are undergoing purifying selection [29]. As shown in Table 1, the Ka/Ks values of all 7 gene pairs were less than 1, indicating that the *ATDUF4228* gene family experienced purifying selection pressure after the duplication events.

**Table 1:** The Ka and Ks values of duplicated *ATDUF4228* gene pairs

Duplicated gene paris	Ka	Ks	Ka/Ks	Duplication type	Types of selection
*AT1G06980/ AT2G30230*	0.16	0.9	0.1778	Segmental	Purify selection
*AT1G10530/ AT1G60010*	0.17	1.17	0.1453	Segmental	Purify selection
*AT1G21010/AT1G76660*	0.22	0.95	0.2316	Segmental	Purify selection
*AT2G23690/ AT4G37240*	0.19	0.83	0.2289	Segmental	Purify selection
*AT3G03280/ AT5G17350*	0.16	1.13	0.1416	Segmental	Purify selection
*AT3G10120/ AT5G03890*	0.3	0.7	0.4286	Segmental	Purify selection
*AT3G50800/ AT5G66580*	0.15	1.39	0.1079	Segmental	Purify selection

The exon-intron organization of the 25 *ATDUF4228* genes was examined to investigate their structural diversity (Additional file 7: Figure S5). Among all 25 *ATDUF4228* genes, only 6 genes exhibited introns, while the other 19 included no introns. Among the 6 genes with introns, the *AT1G71015* gene presented one intron, and *AT5G67620*, *AT5G62900*, *AT5G50090*, *AT1G10530* and *AT1G60010* presented two introns. Interestingly, these 6 genes all belong to group III.

### Expression profiles of *ATDUF4228* genes in different tissues and developmental stages of *A. thaliana*

To investigate the differences in the expression of the *DUF4228* genes in *A. thaliana*, we analysed the expression profiles of the *ATDUF4228* genes in different tissues and at various developmental stages based on microarray data. Among 25 *ATDUF4228* genes, 17 genes already had available microarray data and we could mine their expression data.

**Fig. 5:**
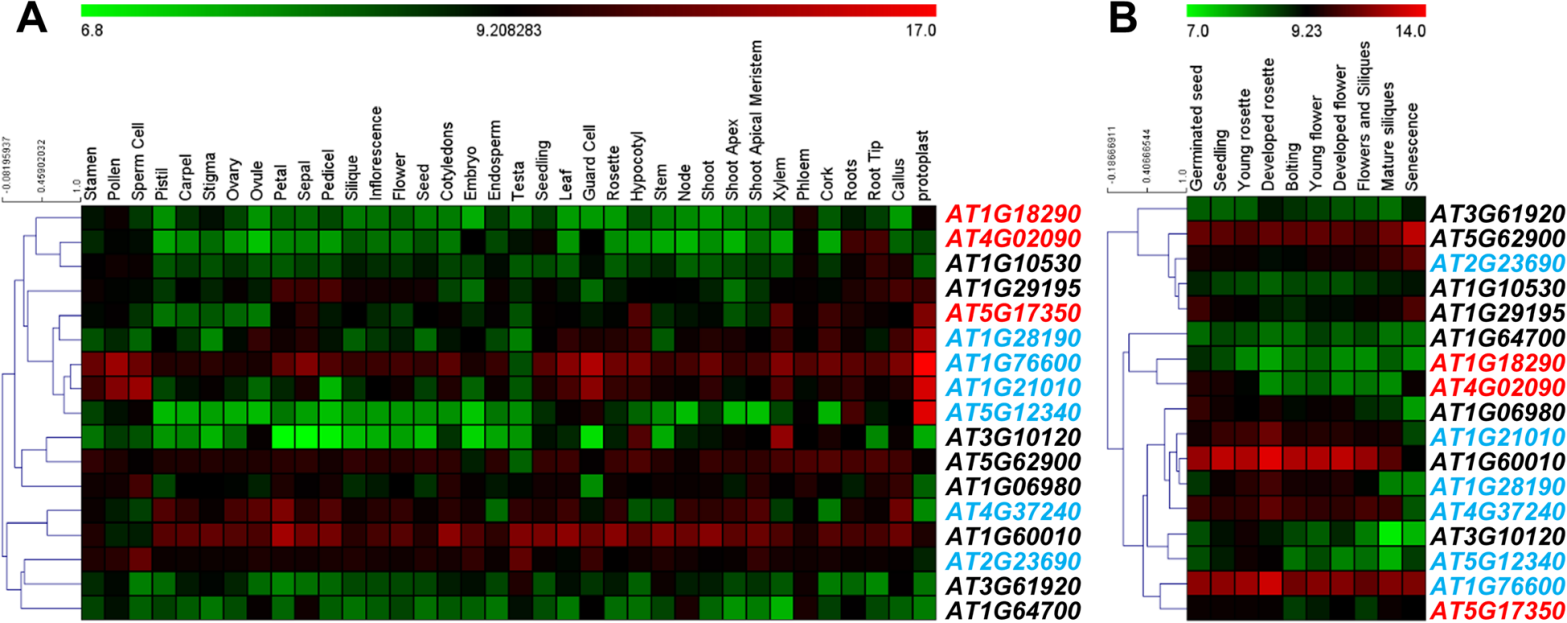
Microarray-based expression pattern of *ATDUF4228* genes in different tissues (**a**) and developmental stages (**b**). The colour bar below the heat maps represents the relative expression values, with white representing the lowest expression while as, brown the highest expression. The gene IDs indicated in red, blue and black belong to group I, group II and group III, respectively

The expression of *ATDUF4228* genes in 36 different tissues was investigated (Fig. 5a). The results showed that the expression levels of the *ATDUF4228* genes presented great variability in different tissues. The expression of some genes was higher in most tissues, such as *AT1G76660*, *AT1G60010* and *AT5G62900*, while others showed lower RNA transcript levels in most tissues, such as *AT5G12340*, *AT1G64700* and *AT1G18290*. Some genes showed higher RNA transcript levels only in certain tissues; for example, the transcript level of *AT4G02090* was highest in the roots and root tips. In some tissues, such as the protoplasts, roots, xylem and calli, there were many genes with high RNA transcript levels.

The RNA transcript levels of *ATDUF4228* genes at 10 different developmental stages were also investigated (Fig. 5b). At most stages of development, the expression of *AT1G76660*, *AT1g60010* and *AT5g62900* was higher than that of the other genes; in contrast, the RNA transcript levels of *AT1G18290* and *AT1g64700* were low at all stages. Several genes were expressed at high levels during specific developmental stages; for instance, *AT1g29195*, *AT2g23690* and *AT5g62900*, were upregulated only at the senescence stage. Distinct expression patterns may imply differences in the function of various *ATDUF4228* genes.

### Stress-related cis-elements in *ATDUF4228* promoters

To further explore the potential regulatory mechanism of *ATDUF4228* genes under plant hormone and abiotic stress stimuli, PlantCARE was used to identify the putative cis-elements in their promoter regions. Nine cis-elements, including five abiotic stress response elements (ARE, cis-element essential for anaerobic induction; MBS, MYB-binding site involved in drought-inducibility; LTR, cis-element related to lowtemperature; DRE, cis-element related to dehydration, low temperature and salt stresses; TC-rich repeats, cis-element involved in defence and stress responsiveness) and four plant hormone-responsive elements (ABRE, cis-element involved in the abscisic acid responsiveness; CGTCAmotif, cis-regulatory element involved in MeJA responsiveness; ERE, ethylene-responsive element; TCA-element, cis-element involved in salicylic acid responsiveness), were analysed and are displayed in Additional file 8: Table S3 and Additional file 9: Figure S6. Overall, cis-elements responsive to abiotic stresses and hormones were widely present in the promoters of the *ATDFU4228* genes, but their distribution was not uniform. The promoters of several genes contained fewer cis-elements, such as *AT1G18290*, *AT1G29195* and *AT5G62900*. Conversely, abiotic stress and plant hormone-responsive cis-elements were abundant in several *ATDUF4228* genes, such as *AT5G66580*, *At1g76660*, *AT3G50800* and *AT1G28190*. The number of cis-acting elements in genes of different subgroups was uneven, and there are more cis-elements in most of the genes belonging to group I than in those of groups II and III. In addition, among the 9 cis-elements, ARE and ABRE were most frequently found in the promoters of *ATDUF4228* genes. The results suggested that the expression of these *ATDFU4228* genes could be regulated by either abiotic stress or stress-related hormones.

### Expression of *ATDUF4228* genes under various abiotic stresses

To investigate the RNA transcript levels of *DUF4228* genes in the roots and aerial parts of *A. thaliana* seedlings, the expression of the *ATDUF4228* genes was detected under osmotic, cold and salt conditions using qRT-PCR. A total of 16 *ATDUF4228* genes were included in the analysis because their expression patterns were consistent across three independent biological replicates.

**Fig. 6:**
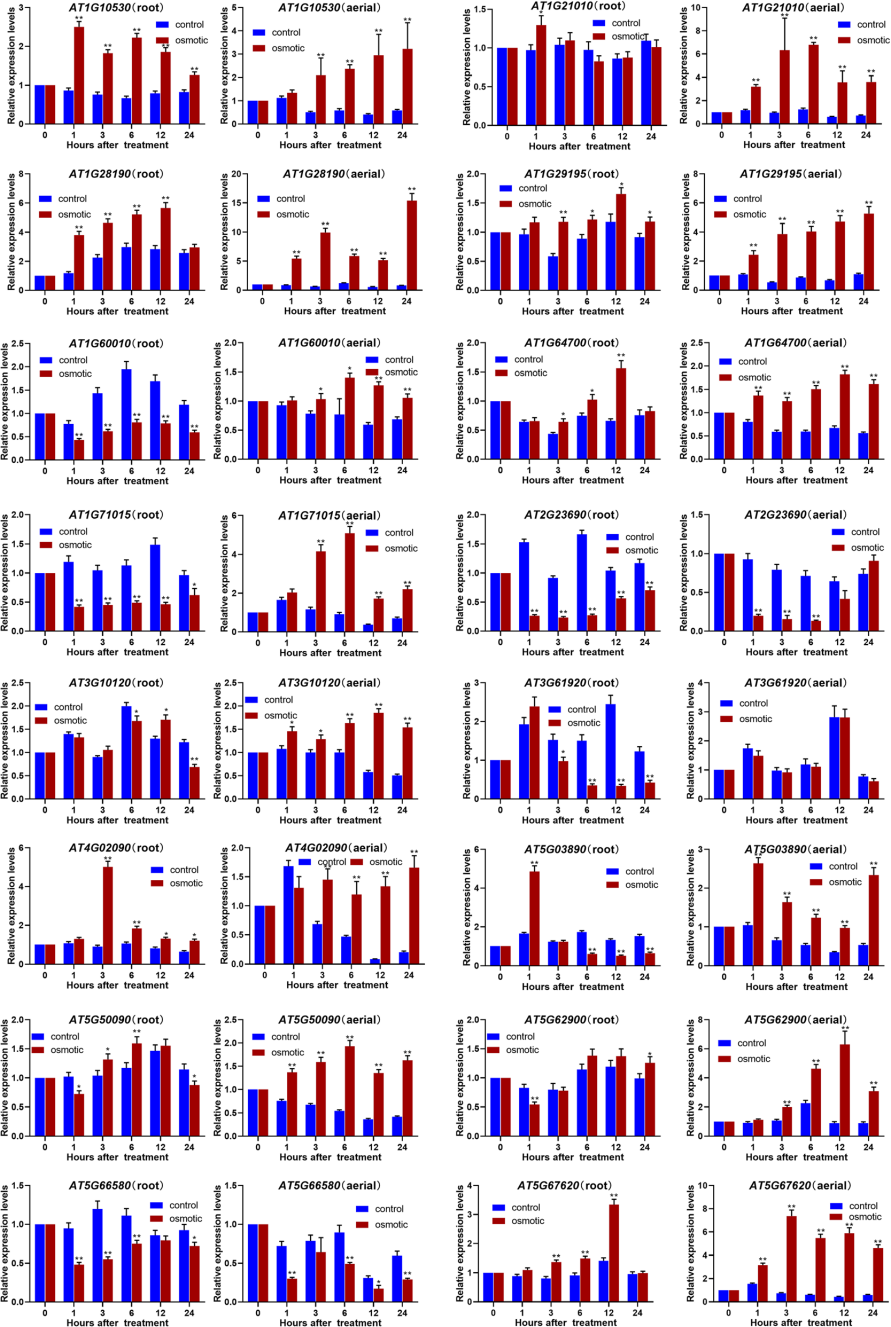
Expression profiling of *ATDUF4228* genes under drought stress. The error bars represent the means of three technical replicates ± SD. Statistically significant differences from the control group are indicated as *P < 0.05; **P < 0.01

qRT-PCR analysis revealed distinct expression patterns among some of the *ATDUF4228* genes in the roots and aerial parts under osmotic stress (Fig. 6). Compared with the control, the RNA transcript levels of some genes were significantly induced or inhibited after osmotic treatment. The RNA transcript levels of *AT1G10530*, *AT1G28190* and *AT5G67620* were increased significantly in both the roots and aerial parts after osmotic treatment. Conversely, the RNA transcript levels of other genes, such as *AT2G23690*, were significantly downregulated. Some genes showed different expression patterns in the roots and aerial parts after treatment. For example, compared to the control, the transcript levels of *AT1G21010* and *AT1G29195* in the roots remained unchanged after treatment, but in aerial tissues, they were upregulated. In contrast, the RNA transcript level of *AT1G71015* was reduced in the roots but increased in aerial parts.

**Fig. 7:**
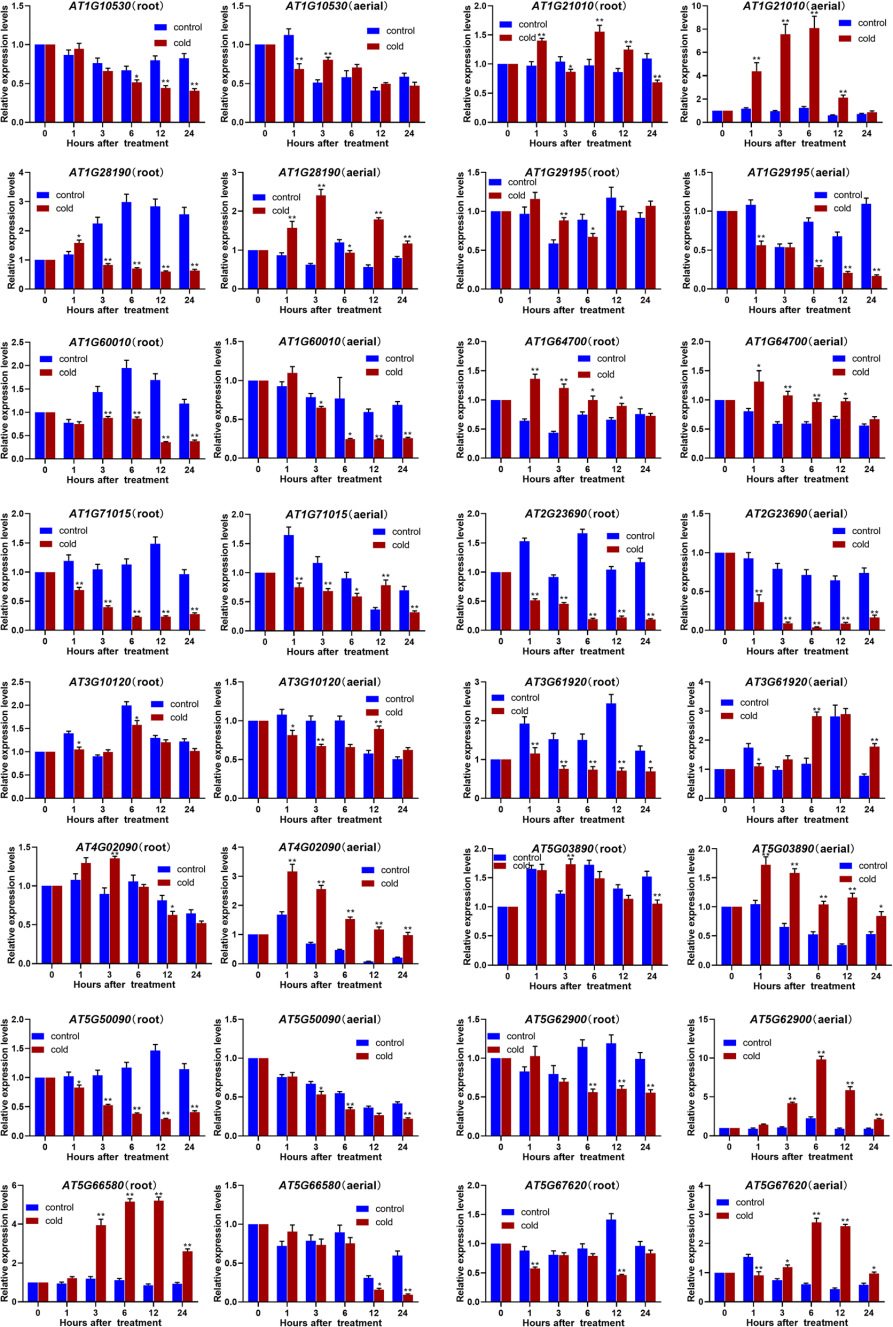
Expression profiling of *ATDUF4228* genes under cold stress. The error bars represent the means of three technical replicates ± SD. Statistically significant differences from the control group are indicated as *P < 0.05; **P < 0.01

The expression patterns of the *ATDUF4228* genes under cold treatment are shown in Fig. 7. The RNA transcript levels of *AT1G21010*, *AT1G28190*, *AT4G02090*, *AT5G62900* and *AT5G67620* in aerial parts were significantly increased after cold treatment. The RNA transcript levels of other genes were significantly downregulated compared to the control, such as *AT1G28190* and *AT1G71015* in the roots, *AT1G29195* in aerial parts, *AT1G60010* and *AT2G23690* in both root and aerial tissues.

**Fig. 8:**
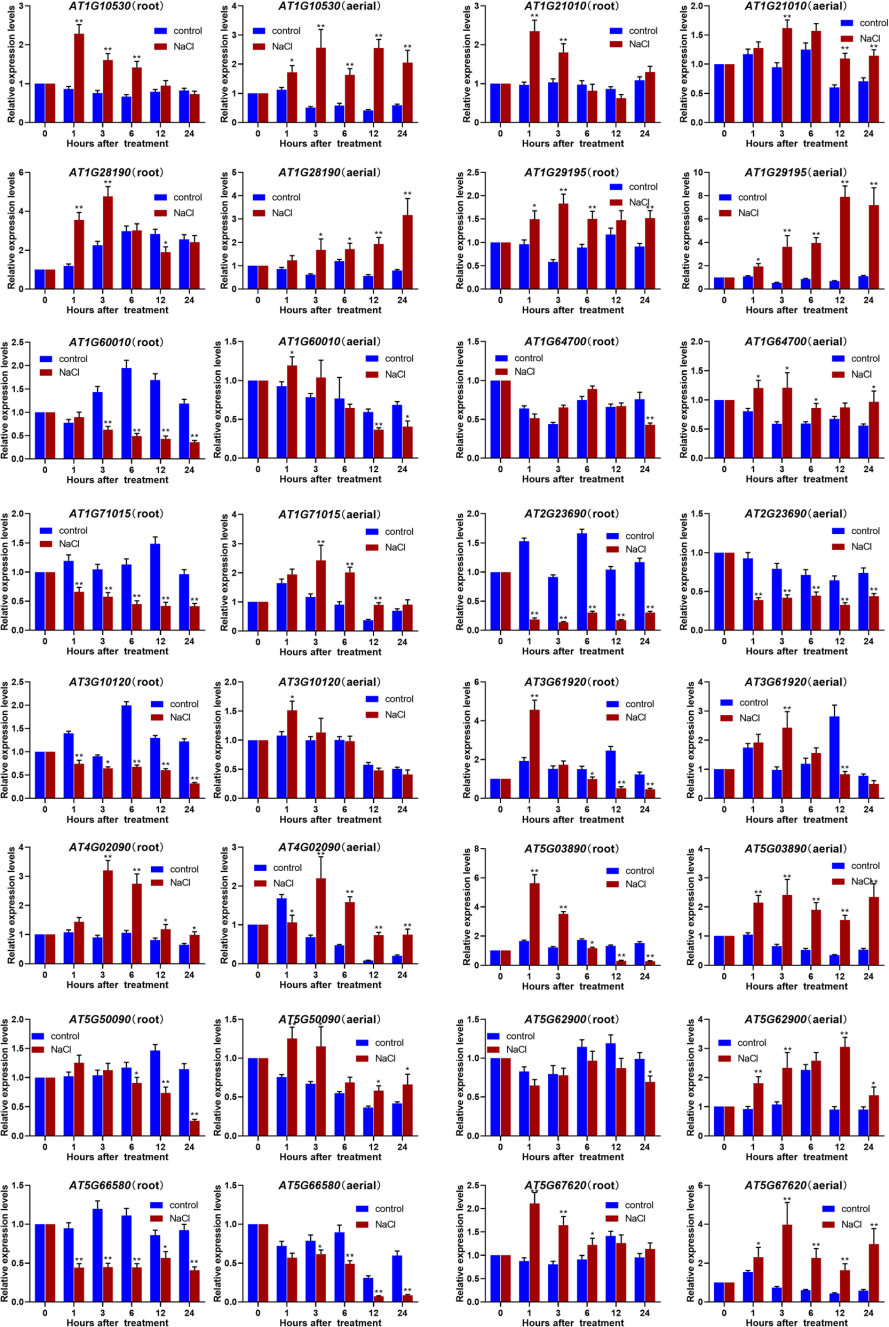
Expression profiling of *ATDUF4228* genes under salt stress. The error bars represent the means of three technical replicates ± SD. Statistically significant differences from the control group are indicated as *P < 0.05; **P < 0.01

The expression patterns of the *ATDUF4228* genes under NaCl treatment were also investigated, and similar to the results under osmotic and cold treatment, different genes presented different expression patterns (Fig. 8). The RNA transcript levels of the *AT1G10530*, *AT4G02090, AT5G03890* and *AT5G67620* genes were significantly upregulated in root and aerial tissues. In contrast, the RNA transcript levels of *AT2G23690* and *AT5G66580* were significantly downregulated after NaCl treatment.

We also found that the expression of some genes, such as *AT4G02090* and *AT5G50090* significantly changed in the control samples (especially in the aerial part). This suggests that the expression of these genes may be regulated by circadian rhythms.

### Protein interaction network of ATDUF4228 proteins

Due to the limited information on the DUF4228 family, protein-protein interaction analysis with other proteins was performed to predict their function in biological processes. In this study, 17 potential protein interaction networks were identified for 25 ATDUF4228 proteins using the STRING search tool with default parameters (Additional file 10: Figure S7) [30]. As shown in Additional file 10: Figure S7, although the predicted networks for the ATDUF4228 proteins were mainly obtained based on text-mining and co-expression analysis, the networks of some of the proteins, such as AT1G10530, AT1G21010, AT1G29195, AT1G60010, AT3G5080, were indeed based on experimental determination or gene fusions.

Several ATDFU4228 proteins interact with some of the abiotic stress-related proteins that have been reported. For example, AT1G21010 and AT1G29195 interact with protein phosphatase 2A; AT1G28190 interacts with WRKY15 and ATL6; AT1G76660 interacts with WRKY40 and CML38; AT5G17350 interacts with AT1G27730 (ZAT10) and AT2G30020 (AP2C1). These interactions indicated that some ATDUF4228 proteins might be involved in abiotic stress tolerance.

## Discussion

Rapid growth of biological sequence databases has accompanied the development of high-throughput genomic and sequencing techniques [7]. At the same time, the fraction of *DUF* gene families in Pfam entries has increased over the past decade [3]. The identification of the biological functions of a large number of *DUF* family genes is a huge challenge. Many studies have shown that genome-wide identification and expression analysis can help researchers to understand the origin, diversity and biological functions of these *DUF* gene families [31, 32].

### Number, expansion and evolution of the *DUF4228* genes in land plants

The distribution and number of the *DUF4228* gene family, encoding domains of unknown function, have not been previously reported in plants. In this study, *DUF4228* genes were identified in 14 land plants but were not present in the two algae (Fig. 1 and Additional file 2: Table S2). This result suggests that the *DUF4228* genes may have originated in land plants. The colonization of the land by plants was one of the most significant events in the history of the Earth [33]. The evolution of land plants involved significant transformations of body plans and genomes [34]. The bryophytes (liverworts, mosses, and hornworts) comprise the earliest-diverging land plant lineages [33, 35]. In this study, *DUF4228* genes were identified in bryophytes but not in algae. This result suggests that the *DUF4228* gene may have arisen when plants colonized the land and could be related to plant adaptation to the terrestrial environment.

The number of *DUF4228* genes in gymnosperms and angiosperms is significantly greater than that in bryophytes and lycophytes, suggesting that the *DUF4228* gene family has undergone expansion during plant evolution. Notably, gymnosperms not only exhibit the greatest abundance of high-confidence *DUF4228* genes among the 14 investigated land plants but also present a much greater number of candidate genes with domain coverage of less than 70% than the other plants (Fig. 1 and Additional file 2: Table S2). The number of *DUF4228* genes in different species is positively correlated with genome size, except in *S. moellendorffii* (Additional file 1: Table S1). We also found that the percentage of *DUF4228* genes among the total number of genes was similar among 12 plant species, with the exception of *S. moellendorffii*. The percentage of *DUF4228* genes in the total number of genes in gymnosperms was not significantly higher than that in angiosperms (Additional file 1: Table S1). Because gymnosperms exhibit a larger genome and greater number of genes than angiosperms, the number of *DUF4228* genes in gymnosperms is significantly higher than that in angiosperms. This expansion of *DUF4228* genes may primarily be associated with the increase in the genome size and the total number of predicted genes. Because of relatively high bootstrap support (98, 100 and 72%, respectively), a total of 489 high-confidence *DUF4228* genes from 14 land plants were divided into three subfamilies through phylogenetic analysis (Fig. 2 and Additional file 3: Figure S1). In contrast to the situation in angiosperms, the *DUF4228* genes arising from the expansion that occurred in gymnosperms mainly belong to groups I and II, whereas the number in group III is not significantly greater (Figs. 1 and 2). The genes belonging to group ΙΙ come only from angiosperms and gymnosperms, and can be clearly divided into two subgroups. Group III can also be divided into two subgroups. Most of the *DUF4228* genes from bryophytes are at the base of each group or subgroup in the evolutionary tree (Fig. 2).

### *ATDUF4228* genes respond to abiotic stresses in *A. thaliana*

To understand the potential biological functions of *DUF4228* genes in plants, the RNA transcript levels of *ATDUF4228* genes after osmotic, cold and salt treatment was further tested in *A. thaliana* (Figs. 6, 7, 8). The *ATDUF4228* genes showing significantly altered expression (fold change≥2) after osmotic, cold and salt treatment are listed in Additional file 11: Table S4. The expression patterns of the different groups *DUF4228* genes presented no obvious regularity. Overall, the number of genes that was upregulated was slightly higher than the number of genes that was downregulated. However, in the roots, a total of 9 *ATDUF4228* genes showed significant changes in expression after cold treatment, 8 of which were downregulated.

Expression analysis revealed that some genes are induced or inhibited by abiotic stress, such as *AT1G10530*, *AT1G21010*, *AT1G28190*, *AT1G29195*, *AT1G60010*, *AT1G71015*, *AT2G23690*, *AT4G02090*, *AT5G03890 AT5G62900*, *AT5G66580* and *AT5G67620* (Figs. 6, 7, 8 and Additional file 11: Table S4). Amongthese genes, *AT1G71015* is homologous to *MsDUF* (JX183734) which has been reported in *Medicago sativa*, and its expression level is regulated by cold and drought [25].

In addition, the investigation of cis-acting elements in promoters and protein-protein interaction analysis can also provide clues for further studying the biological functions of genes. The *ATDUF4228* genes, that not only responded to abiotic stresses but are also predicted to interact with stress-related proteins were selected for analysis.

The RNA transcript levels of *AT1G21010* and *AT1G29195* changed significantly after abiotic stress. Under cold and osmotic stress, the expression of the *AT1G21010* gene was significantly upregulated in aerial parts (Figs. 6, 7). The expression of the *AT1G29195* gene in aerial tissues was upregulated under salt stress and osmotic stress, while its expression was significantly downregulated under cold stress (Figs. 6, 7, 8). Protein-protein interaction analysis showed that AT1G21010 and AT1G29195 may participate in PP2A-mediated pathways through interaction with ATB’ DELTA (AT3G26030, protein phosphatase 2A regulatory subunit isoform B′ delta) and PP2AA3 (AT1G13320), respectively (Additional file 10: Figure S7). Many reports have shown that PP2A plays important roles in biotic and abiotic stress signalling pathways in plants [36–38], implying that AT1G21010 and AT1G29195 may be involved in the response to abiotic stress in plants through interaction with PP2A. In addition, there are 4 ABRE cis-acting elements in the promoter of *AT1G21010*, indicating that it may be associated with an ABA-dependent signalling pathway (Additional file 9: Figure S6). Another *DUF4228* gene in *A. thaliana*, *AT1G10530*, was found to be induced by osmotic and NaCl and to interact with ATB’ DELTA, similar to *AT1G21010*.

The RNA transcript levels of *AT5G67620* in aerial tissues was increased under osmotic, cold and NaCl treatment (Figs. 6, 7, 8). Protein-protein interaction analysis showed that AT5G67620 interacts with two proteins, AT4G24840 and AT3G08530 (Additional file 10: Figure S7), and the interaction between them has been verified with yeast two-hybrid array [39]. AT3G08530, a CHC2 heavy chain subunit of clathrin, is involved in vesicle-mediated trafficking. A mutant of AT3G08530 shows increased drought tolerance due to defects in stomatal movement [40]. These findings indicate that At5G67620 may participate in the movement of the stomata via interacting with AT3G08530.

The RNA transcript levels of *AT1G28190* was increased after the osmotic, cold and NaCl treatments (except in the roots after cold treatment) (Figs. 6, 7, 8). Several proteins that interact with AT1G28190 are related to plant stress resistance (Additional file 10: Figure S7). For example, ATMYB15 (AT2G23320) modulates plant growth and salt/osmotic stress responses [41]. ATL6 (AT3G05200) is a key component of both C/N regulation and the defence response and is involved in the immune response system in *A. thaliana* [42]. XLG2 (AT4G34390) acts as a positive regulator of resistance to pathogens that triggers the salicylic acid pathway [43]. Four TCA elements, involved in salicylic acid responsiveness, were found in the promoter region of *AT1G28190*, which was the greatest number among the 25 *ATDUF4228* genes (Additional file 9: Figure S6). These results suggest that *AT1G28190* may be involved in the signalling pathway of the salicylic acid response.

In addition to controlling abiotic stress responses, the analysis of protein-protein interactions revealed that some *ATDUF4228* genes may be involved in the growth and development of plants. For example, as shown in Additional file 10: Figure S7, AT1G60010 interacts with BBX16 (COL7, AT1G73870). BBX16 is a critical factor linking light perception to changes in auxin levels in *A. thaliana* and increases branching number under a high R:FR [44, 45].

The results of this study shed light the potentially important role played by *DUF4228* genes in plant resistance to abiotic stress.

## Conclusions

In this study, 489 *DUF4228* genes were identified in 14 high-quality genomes of land plants for the first time, and a comprehensive analysis of phylogenetic relationships and conserved motifs was carried out. The 489 *DUF4228* genes can be divided into three groups, among which group II may be unique to Spermatophyta.

A total of 25 *DUF4228* genes were identified in *A. thaliana*. Furthermore, the chromosomal locations, gene duplications, cis-elements and expression patterns of the *ATDUF4228* gene family members were investigated. According to analyses of expression, protein interactions and responsive cis-elements, some *DUF4228* genes in *A. thaliana* may be related to the plant responses to abiotic stress, such as *AT1G21010*, *AT1G28190*, *AT1G29195* and *AT5G67620*.

## Methods

### Data retrieval and identification of *DUF4228* genes

In this study, we identified *DUF4228* candidate genes from 16 representative plants. The protein databases were downloaded from the Phytozome12 [46] (*Volvox carteri*, *Chlamydomonas reinhardtii*, *Marchantia polymorpha*, *Physcomitrella patens*, *Selaginella moellendorffii*, *Amborella trichopoda*, *Oryza sativa*, *Brachypodium distachyon*, *Zea mays*, *Medicago truncatula*, *Eucalyptus grandis*, *Populus trichocarpa*, *Aquilegia coerulea*, and *Arabidopsis thaliana*) and ConGenIE [47] (*Picea abies* and *Pinus taeda*). Detailed genomic information for the 16 plants included in this study is listed in Additional file 1: Table S1.

To identify the *DUF4228* gene family members in plants, a profile hidden Markov model (pHMM) of the DUF4228 (Pfam: PF14009) domain was downloaded from Pfam 31.0 [48]. Hmmsearch (HMMer package version3.1b1) was used to search DUF4228.hmm against the protein sequences from each plant genome [49]. To ensure search reliability, domain hits beyond the gathering threshold (E-value 1e^− 10^) were filtered out before downstream analysis. For gene loci with multiple predicted isoforms, the primary isoform was used if the primary isoform annotation was available; otherwise, the longest protein was used. The Pfam databases were employed to confirm DUF4228 domains in the candidate proteins. To ensure the search reliability, domain hits beyond the gathering threshold (E-value 1e^− 10^) and those with less than 70% coverage were filtered out manually before downstream analysis.

The Compute pI/MW tool of the ExPASy server (http://web.expasy.org) was used to calculate the molecular weight (MW) and theoretical isoelectric point (pI) of the DUF proteins [50]. The WoLF PSORT program (https://wolfpsort.hgc.jp/) was used to predict protein subcellular localization [51].

### Phylogenetic analysis and conserved motif analysis

The amino acid sequences of the *DUF4228* genes identified from 14 plants were subjected to alignment by ClustalW with default settings [52]. Phylogenetic analyses were conducted by using the maximum likelihood (ML) method. First, the ML estimation used the best-fitting model of sequence evolution as determined by ModelFinder [53]. Then, IQ-TREE was used to infer the ML tree with 1000 bootstraps replicates for alignment [54, 55]. All phylogenetic trees were edited and displayed with the online tool iTOL [56].

MEME software v5.0.5 (http://memesuite.org/tools/meme) was employed to identify conserved motifs with the default parameters, except that the maximum number of motifs was set to 10 [27]. The map was redrawn with Tbtools [57].

### Chromosomal distribution, gene duplication and gene structure

Circos (version 0.63) (http://circos.ca/) was used to visualize the chromosomal locations and synteny relationships of *ATDUF4228* genes from *A. thaliana* [58]. The Multiple Collinearity Scan toolkit (MCScanX) (http://chibba.pgml.uga.edu/mcscan2/) was adopted to analyse gene duplication events [28]. The non-synonymous (Ka) and synonymous (Ks) substitutions of each duplicated *ATDUF4228* genes was obtained from PGDD [59]. The exon/intron organization of the *ATDUF4228* genes was visualized by comparing the cDNA sequences with their corresponding genomic DNA sequences using the Gene Structure Display Server 2.0 (GSDS, http://gsds.cbi.pku.edu.cn) [60].

### Analysis of cis-elements in *ATDUF4228* gene promoters

The upstream sequences (2000 bp) of the *ATDUF4228*-coding sequences were retrieved from the *A. thaliana* Information Resource (TAIR) and then submitted to the PlantCARE program (http://bioinformatics.psb.ugent.be/webtools/plantcare/html/) [61]. Nine putative cis-elements were investigated in this study, and the mapping of their distribution in the promoters of the *ATDUF4228* genes was illustrated using IBS1.0.3 [62].

### Gene expression analysis with microarray data

The microarray-based expression profiles of the *ATDUF4228* genes were retrieved from publicly available Affymetrix microarray data. Expression patterns were mined from 29 different tissues and 10 developmental stages of *A. thaliana* by retrieving the log_2_-transformed Affymetrix data from the respective arrays and generating a heatmap using the Genevestigator database tool [63].

### Abiotic stress treatments, RNA isolation and qRT-PCR

The methods for the preparation of the *A. thaliana* (Col-0) seedlings referred to previous studies with minor modifications [64, 65]. The wild-type seeds were sown on ½MS plates with 0.75% agar. After stratification for 3 days at 4 °C, the plates were transferred to a plant culture room (16 h/8 h day/night cycles at 22 °C) for the following abiotic stress treatments.

For cold stress, different sets of plates containing 14-day-old seedlings were placed in incubators set at 4 °C for 0, 1, 3, 6, 12 or 24 h. For osmotic or salinity treatment, the 14-day-old seedlings were transferred to ½MS liquid medium supplemented with 150 mM NaCl (salt stress) or 300 mM mannitol (osmotic stress), and the different stresses were applied for 24 h. A fresh medium-only control was conducted in parallel. Samples of the stress-treated and non-stressed plants were collected at the indicated times after 3 h of light exposure. After treatment, the roots and the aerial parts of the seedlings were harvested separately, and 40 *A. thaliana* seedlings were randomly mixed at each treatment time and frozen immediately into liquid nitrogen.

qRT-PCR was performed according to our previously reported methods [66]. In brief, total RNA was isolated using a total RNA extraction kit (Tiangen, China). The quality and quantity of the RNA was evaluated by using agarose gel electrophoresis and a Quawell micro volume spectrophotometer (Q5000, USA), respectively. Then, 1 μg of total RNA after DNase I digestion was reverse transcribed into cDNA using the PrimeScript™ II 1st Strand cDNA Synthesis Kit (TaKaRa, Japan). The cDNA was amplified using a QuantiNova SYBR Green PCR Kit (QIAGEN, Germany) with a Roche LightCycler 480 Real Time PCR system (Roche, Switzerland). The thermal cycling program was 95 °C for 2 m, followed by 40 cycles of 95 °C for 5 s, and 60 °C for 10 s. The melting curves were analysed at 60–95 °C after 40 cycles. All qRT-PCR assays were carried out with three technical replicates. The relative RNA transcript levels of the *ATDUF4228* genes were calculated according to the 2^−ΔΔCT^ method [67]. The *ATEF1ɑ* (*AT5G60390*) gene was chosen as the reference gene for the qRT-PCR analysis. All primers used in this study are listed in (Additional file 12: Table S5). Each entire experiment was repeated three times independently. The *ATDUF4228* genes with consistent expression patterns across three independent biological replicates were included in the subsequent analysis.

### Protein-protein interaction analysis

Because some *ATDUF4228* genes were found to be induced or inhibited by various abiotic stresses, we were interested in determining their interactions with other genes. The Protein-protein interaction (PPI) networks were identified in *A. thaliana* using the STRING search tool (version11.0) (https://string-db.org/) with default parameters [30].

## Supplementary information

**Additional file 1: Table S1.** Information on the species and their genomes used for *DUF4228* gene identification.

**Additional file 2: Table S2.** List of *DUF4228* genes included in this study and the features of their encoded proteins. Those DUF4228 proteins with a domain coverage of less than 70% are labelled in yellow. The genes are marked in red because some amino acid residues in their protein sequence are represented by “X”.

**Additional file 3: Figure S1.** Details of the phylogenetic classification of *DUF4228* genes in land plant lineages. Phylogenetic tree was constructed using the ML method with IQ-tree. Green lines represent bryophytes, pink lines represent pteridophytes, blue lines represent gymnosperms and red lines represent angiosperms.

**Additional file 4: Figure S2.** Distribution of the DUF4228 domain in the DUF4228 proteins of plants.

**Additional file 5: Figure S3.** The motif compositions of DUF4228 proteins in plants. Motifs conserved across DUF4228 proteins were identified through MEME analysis.

**Additional file 6: Figure S4.** The distribution of *ATDUF4228* genes on the chromosomes. The chromosomal position of each *ATDUF4228* gene was mapped according to the genome of *A. thaliana*. The chromosome number is indicated at the top of each chromosome.

**Additional file 7: Figure S5.** Exon-intron structure of the *ATDUF4228* genes. Yellow boxes: exons; blue boxes: UTRs; black lines: introns. The lengths of the boxes and lines are scaled based on gene length.

**Additional file 8: Table S3.** Number and location of abiotic stress- and hormone-responsive cis-elements present in the promoter regions of *ATDUF4228* genes.

**Additional file 9: Figure S6.** Promoter analyses of *ATDUF4228* genes. The promoter sequences (2 kb upstream of ATG) of the 25 *ATDUF4228* genes were analysed by PlantCARE. Cis-elements responsive to abiotic stresses and plant hormones are indicated in different colours and shapes.

**Additional file 10: Figure S7.** Protein-protein interaction network of ATDUF4228 proteins. In the network generated by STRINGV9.1, each node represents a protein and each edge represents an interaction, coloured by evidence type.

**Additional file 11: Table S4.** Summary of *ATDUF4228* genes with significantly altered expression (fold change≥2) after osmotic, cold and salt treatment. “up” indicates upregulated expression with a minimum 2-fold change compared with the control. “down” indicates downregulated expression with a minimum 2-fold change compared with the control. “-” indicates no obvious change detected.

**Additional file 12: Table S5.** All primers used in this study.

## Data Availability

The genome databases were downloaded from the Phytozome12 (https://phytozome.jgi.doe.gov/pz/portal.html) and the ConGenIE (http://congenie.org/). The datasets supporting the conclusions of this article are included in the article and its Additional files.

## Abbreviations

*A. thaliana*: *Arabidopsis thaliana*
ABA: Abscisic acid
Chr: Chromosomes
DUF: Domain of unknown function
EF1α: Elongation factor 1α
HMM: Hidden Markov Model
Ka: Non-synonymous
Ks: Synonymous
ML: Maximum Likelihood
MW: Molecular weight
*O. sativa*: *Oryza sativa*
PI: Isoelectric point
qRT-PCR: Quantitative reverse transcription polymerase chain reaction
*S. moellendorffii*: *Selaginella moellendorffii*

## References

[1] Bateman A, Coggill P, Finn RD (2010). DUFs: families in search of function. Acta Crystallogr Sect F Struct Biol Cryst Commun.

[2] Schultz J, Milpetz F, Bork P, Ponting CP (1998). SMART, a simple modular architecture research tool: identification of signaling domains. Proc Natl Acad Sci U S A.

[3] Bateman A, Smart A, Luciani A, Salazar GA, Mistry J, Richardson LJ, Qureshi M, El-Gebali S, Potter SC, Finn RD (2018). The Pfam protein families database in 2019. Nucleic Acids Res.

[4] Punta M, Coggill PC, Eberhardt RY, Mistry J, Tate J, Boursnell C, Pang N, Forslund K, Ceric G, Clements J (2012). The Pfam protein families database. Nucleic Acids Res.

[5] Mewalal R, Mizrachi E, Coetzee B, Mansfield SD, Myburg AA (2016). The Arabidopsis domain of unknown function 1218 (DUF1218) containing proteins, MODIFYING WALL LIGNIN-1 and 2 (At1g31720/MWL-1 and At4g19370/MWL-2) function redundantly to alter secondary cell wall lignin content. PLoS One.

[6] Li J, Hu E, Chen X, Xu J, Lan H, Li C, Hu Y, Lu Y (2016). Evolution of DUF1313 family members across plant species and their association with maize photoperiod sensitivity. Genomics.

[7] Mudgal R, Sandhya S, Chandra N, Srinivasan N (2015). De-DUFing the DUFs: deciphering distant evolutionary relationships of domains of unknown function using sensitive homology detection methods. Biol Direct.

[8] Wang L, Shen R, Chen L-T, Liu Y-G (2014). Characterization of a novel DUF1618 gene family in rice. J Integr Plant Biol.

[9] Hou C, Tian W, Kleist T, He K, Garcia V, Bai F, Hao Y, Luan S, Li L (2014). DUF221 proteins are a family of osmosensitive calcium-permeable cation channels conserved across eukaryotes. Cell Res.

[10] Li LH, Lv MM, Li X, Ye TZ, He X, Rong SH, Dong YL, Guan Y, Gao XL, Zhu JQ (2018). The rice OsDUF810 family: OsDUF810.7 may be involved in the tolerance to salt and drought. Mol Biol (Mosk).

[11] Li L, Xie C, Ye T, Xu J, Chen R, Gao X, Zhu J, Xu Z (2017). Molecular characterization, expression pattern and function analysis of the rice OsDUF866 family. Biotechnol BiotecI Eq.

[12] Li L, Ye T, Guan Y, Lv M, Xie C, Xu J, Gao X, Zhu J, Cai L, Xu Z (2018). Genome-wide identification and analyses of the rice OsDUF936 family. Biotechnol BiotecI Eq.

[13] Cao X, Yang K-Z, Xia C, Zhang X-Q, Chen L-Q, Ye D (2009). Characterization of DUF724 gene family in Arabidopsis thaliana. Plant Mol Biol.

[14] Nietzsche M, Schiessl I, Bornke F (2014). The complex becomes more complex: protein-protein interactions of SnRK1 with DUF581 family proteins provide a framework for cell- and stimulus type-specific SnRK1 signaling in plants. Front Plant Sci.

[15] Xin Z, Mandaokar A, Chen J, Last RL, Browse J (2007). Arabidopsis ESK1 encodes a novel regulator of freezing tolerance. Plant J.

[16] Kim SJ, Ryu MY, Kim WT (2012). Suppression of Arabidopsis RING-DUF1117 E3 ubiquitin ligases, AtRDUF1 and AtRDUF2, reduces tolerance to ABA-mediated drought stress. Biochem Biophys Res Commun.

[17] Guo C, Luo C, Guo L, Li M, Guo X, Zhang Y, Wang L, Chen L (2016). OsSIDP366, a DUF1644 gene, positively regulates responses to drought and salt stresses in rice. J Integr Plant Biol.

[18] Cui Y, Wang M, Zhou H, Li M, Huang L, Yin X, Zhao G, Lin F, Xia X, Xu G (2016). OsSGL, a novel DUF1645 domain-containing protein, confers enhanced drought tolerance in transgenic rice and Arabidopsis. Front Plant Sci.

[19] Li M, Guo L, Guo C, Wang L, Chen L (2016). Over-expression of a DUF1644 protein gene, SIDP361 , enhances tolerance to salt stress in transgenic rice. J Plant Biol.

[20] Luo C, Guo C, Wang W, Wang L, Liang C (2013). Overexpression of a new stress-repressive gene OsDSR2 encoding a protein with a DUF966 domain increases salt and simulated drought stress sensitivities and reduces ABA sensitivity in rice. Plant Cell Rep.

[21] Albornos L, Martín I, Labrador E, Dopico B (2017). Three members of Medicago truncatula ST family are ubiquitous during development and modulated by nutritional status (MtST1) and dehydration (MtST2 and MtST3). BMC Plant Biol.

[22] Hou X, Liang Y, He X, Shen Y, Huang Z (2013). A novel ABA-responsive TaSRHP gene from wheat contributes to enhanced resistance to salt stress in Arabidopsis thaliana. Plant Mol Biol Rep.

[23] Gu L, Cheng H (2014). Isolation, molecular cloning and characterization of a cold-responsive gene, AmDUF1517, from Ammopiptanthus mongolicus. Plant Cell Tiss Org.

[24] Gao Q, Li X, Jia J, Zhao P, Liu P, Liu Z, Ge L, Chen S, Qi D, Deng B (2016). Overexpression of a novel cold-responsive transcript factor LcFIN1 from sheepgrass enhances tolerance to low temperature stress in transgenic plants. Plant Biotechnol J.

[25] Wang Y, Zhang Z, Liu H, An Y, Han B, Wu Y, Chang L, Hu T, Yang P (2018). Overexpression of an alfalfa ( *Medicago sativa* ) gene, MsDUF , negatively impacted seed germination and response to osmotic stress in transgenic tobacco. Plant Cell Tiss Org.

[26] Dong W, Vannozzi A, Chen F, Hu Y, Chen Z, Zhang L (2018). MORC domain definition and evolutionary analysis of the MORC gene family in green plants. Genome Biol Evol.

[27] Bailey TL, Boden M, Buske FA, Frith M, Grant CE, Clementi L, Ren J, Li WW, Noble WS (2009). MEME Suite: tools for motif discovery and searching. Nucleic Acids Res.

[28] Wang Y, Tang H, DeBarry JD, Tan X, Li J, Wang X, Lee T-H, Jin H, Marler B, Guo H (2012). MCScanX: a toolkit for detection and evolutionary analysis of gene synteny and collinearity. Nucleic Acids Res.

[29] Lynch M, Conery JS (2000). The evolutionary fate and consequences of duplicate genes. Science.

[30] Szklarczyk D, Franceschini A, Wyder S, Forslund K, Heller D, Huerta-Cepas J, Simonovic M, Roth A, Santos A, Tsafou KP (2015). STRING v10: protein-protein interaction networks, integrated over the tree of life. Nucleic Acids Res.

[31] Chang YL, Li WY, Miao H, Yang SQ, Li R, Wang X, Li WQ, Chen KM (2016). Comprehensive genomic analysis and expression profiling of the NOX gene families under abiotic stresses and hormones in plants. Genome Biol Evol.

[32] Moturu TR, Thula S, Singh RK, Nodzynski T, Varekova RS, Friml J, Simon S (2018). Molecular evolution and diversification of the SMXL gene family. J Exp Bot.

[33] Arteaga-Vazquez MA (2016). Land plant evolution: listen to your elders. Curr Biol.

[34] Ishizaki K (2017). Evolution of land plants: insights from molecular studies on basal lineages. Biosci Biotechnol Biochem.

[35] Kenrick P, Crane PR (1997). The origin and early evolution of plants on land. Nature.

[36] Hu R, Zhu Y, Shen G, Zhang H (2017). Overexpression of the PP2A-C5 gene confers increased salt tolerance in Arabidopsis thaliana. Plant Signal Behav.

[37] Rahikainen Moona, Pascual Jesús, Alegre Sara, Durian Guido, Kangasjärvi Saijaliisa (2016). PP2A Phosphatase as a Regulator of ROS Signaling in Plants. Antioxidants.

[38] Pernas M, Garcia-Casado G, Rojo E, Solano R, Sanchez-Serrano JJ (2007). A protein phosphatase 2A catalytic subunit is a negative regulator of abscisic acid signalling. Plant J.

[39] Dreze M, Carvunis AR, Charloteaux B, Galli M, Pevzner SJ, Tasan M, Ahn YY, Balumuri P, Barabási AL, Bautista V (2011). Evidence for network evolution in an Arabidopsis interactome map. Science.

[40] Larson ER, Van ZE, Roux C, Marion-Poll A, Blatt MR (2017). Clathrin heavy chain subunits coordinate endo- and exocytic traffic and affect stomatal movement. Plant Physiol.

[41] Vanderauwera S, Vandenbroucke K, Inze A, van de Cotte B, Muhlenbock P, De Rycke R, Naouar N, Van Gaever T, Van Montagu MC, Van Breusegem F (2012). AtWRKY15 perturbation abolishes the mitochondrial stress response that steers osmotic stress tolerance in Arabidopsis. Proc Natl Acad Sci U S A.

[42] Maekawa S, Sato T, Asada Y, Yasuda S, Yoshida M, Chiba Y, Yamaguchi J (2012). The Arabidopsis ubiquitin ligases ATL31 and ATL6 control the defense response as well as the carbon/nitrogen response. Plant Mol Biol.

[43] Maruta N, Trusov Y, Brenya E, Parekh U, Botella JR (2015). Membrane-localized extra-large G proteins and Gbg of the heterotrimeric G proteins form functional complexes engaged in plant immunity in Arabidopsis. Plant Physiol.

[44] Wang H, Zhang Z, Li H, Zhao X, Liu X, Ortiz M, Lin C, Liu B (2013). CONSTANS-LIKE 7 regulates branching and shade avoidance response in Arabidopsis. J Exp Bot.

[45] Zhang Z, Ji R, Li H, Zhao T, Liu J, Lin C, Liu B (2014). CONSTANS-LIKE 7 (COL7) is involved in phytochrome B (phyB)-mediated light-quality regulation of auxin homeostasis. Mol Plant.

[46] Goodstein DM, Shu S, Russell H, Rochak N, Hayes RD, Joni F, Therese M, William D, Uffe H, Nicholas P (2012). Phytozome: a comparative platform for green plant genomics. Nucleic Acids Res.

[47] Sundell D, Mannapperuma C, Netotea S, Delhomme N, Lin Y-C, Sjödin A, Van de Peer Y, Jansson S, Hvidsten TR, Street NR (2015). The plant genome integrative explorer resource: PlantGenIE.org. New Phytol.

[48] Finn RD, Coggill P, Eberhardt RY, Eddy SR, Mistry J, Mitchell AL, Potter SC, Punta M, Qureshi M, Sangrador-Vegas A (2016). The Pfam protein families database: towards a more sustainable future. Nucleic Acids Res.

[49] Eddy SR (1998). Profile hidden Markov models. Bioinformatics..

[50] Walker JM. The proteomics protocols handbook. Totowa: Humana press; 2005.

[51] Horton P, Park KJ, Obayashi T, Fujita N, Harada H, Adamscollier CJ, Nakai K (2007). WoLF PSORT: protein localization predictor. Nucleic Acids Res.

[52] Larkin MA, Blackshields G, Brown NP, Chenna R, McGettigan PA, McWilliam H, Valentin F, Wallace IM, Wilm A, Lopez R (2007). Clustal W and Clustal X version 2.0. Bioinformatics.

[53] Kalyaanamoorthy S, Minh BQ, Wong TKF, von Haeseler A, Jermiin LS (2017). ModelFinder: fast model selection for accurate phylogenetic estimates. Nat Methods.

[54] Trifinopoulos J, Nguyen L-T, von Haeseler A, Minh BQ (2016). W-IQ-TREE: a fast online phylogenetic tool for maximum likelihood analysis. Nucleic Acids Res.

[55] Nguyen L-T, Schmidt HA, von Haeseler A, Minh BQ (2014). IQ-TREE: a fast and effective stochastic algorithm for estimating maximum-likelihood phylogenies. Mol Biol Evol.

[56] Letunic I, Bork P (2007). Interactive tree of life (iTOL): an online tool for phylogenetic tree display and annotation. Bioinformatics.

[57] Chen C, Xia R, Chen H, He Y. TBtools, a Toolkit for Biologists integrating various biological data handling tools with a user-friendly interface. BioRxiv Preprint Mar. 2018;289660.

[58] Krzywinski M, Schein JIJGR (2009). Circos: an information aesthetic for comparative genomics. Genome Res.

[59] Lee T-H, Tang H, Wang X, Paterson AH (2013). PGDD: a database of gene and genome duplication in plants. Nucleic Acids Res.

[60] Guo A-Y, Hu B, Gao G, Zhang H, Luo J, Jin J (2014). GSDS 2.0: an upgraded gene feature visualization server. Bioinformatics.

[61] Lescot M, Dehais P, Thijs G, Marchal K, Moreau Y, Van de Peer Y, Rouze P, Rombauts S (2002). PlantCARE, a database of plant cis-acting regulatory elements and a portal to tools for in silico analysis of promoter sequences. Nucleic Acids Res.

[62] Liu W, Xie Y, Ma J, Luo X, Nie P, Zuo Z, Lahrmann U, Zhao Q, Zheng Y, Zhao Y (2015). IBS: an illustrator for the presentation and visualization of biological sequences. Bioinformatics.

[63] Hruz Tomas, Laule Oliver, Szabo Gabor, Wessendorp Frans, Bleuler Stefan, Oertle Lukas, Widmayer Peter, Gruissem Wilhelm, Zimmermann Philip (2008). Genevestigator V3: A Reference Expression Database for the Meta-Analysis of Transcriptomes. Advances in Bioinformatics.

[64] Barciszewska-Pacak M, Milanowska K, Knop K, Bielewicz D, Nuc P, Plewka P, Pacak AM, Vazquez F, Karlowski W, Jarmolowski A (2015). Arabidopsis microRNA expression regulation in a wide range of abiotic stress responses. Front Plant Sci.

[65] Kilian J, Whitehead D, Horak J, Wanke D, Weinl S, Batistic O, D'Angelo C, Bornberg-Bauer E, Kudla J, Harter K (2007). The AtGenExpress global stress expression data set: protocols, evaluation and model data analysis of UV-B light, drought and cold stress responses. Plant J.

[66] Qi Y, Liu K, Niu X, Qi W, Wan Y, Yang F, Li G, Wang Y, Wang R (2018). Genome-wide identification of PP2C genes and their expression profiling in response to drought and cold stresses in Medicago truncatula. Sci Rep.

[67] Livak KJ, Schmittgen TD (2001). Analysis of relative gene expression data using real-time quantitative PCR and the 2(−Delta Delta C(T)) method. Methods.

