# Supplementary material for: Comprehensive genomic analysis of the DUF4228 gene family in land plants and expression profiling of ATDUF4228 under abiotic stresses
Source: BMC Genomics. 2020 Jan 3;21:12. doi: 10.1186/s12864-019-6389-3 (PMC6942412; doi:10.1186/s12864-019-6389-3)
Supplement: Supplementary file 1 — Additional file 1: Table S1. Information on the species and their genomes used for DUF4228 gene identification. [file 12864_2019_6389_MOESM1_ESM.docx]

**Table S1** Information on the species and their genomes used for *DUF4228* gene identification

| Lineage | Organism | Genome size | Number of predicted genes | Number of *DUF4228* genes | ‰UGTs in total number of genes |
| --- | --- | --- | --- | --- | --- |
| Alage | *Volvox carteri* | 131.2 Mb | 14,247 | 0 | 0 |
|  | *Chlamydomonas reinhardtii* | 111.1 Mb | 17,741 | 0 | 0 |
| Moss | *Marchantia polymorpha* | 225.8 Mb | 19,287 | 15 | 0.78 |
|  | *Physcomitrella patens* | 473 Mb | 32,926 | 18 | 0.54 |
| Lycophytes | *Selaginella moellendorffii* | 212.5 Mb | 22,273 | 3 | 0.13 |
| Gymnosperms | *Picea abies* | 19.6Gb | 91,045 | 91 | 1 |
|  | *Pinus taeda* | 22 Gb | 66,632 | 65 | 0.98 |
| Basal angiosperms | *Amborella trichopoda* | 706 Mb | 26,846 | 21 | 0.78 |
| Monocots | *Oryza sativa* | 372 Mb | 42,189 | 34 | 0.80 |
|  | *Brachypodium distachyon* | 272 Mb | 36,647 | 35 | 0.96 |
|  | *Zea mays* | 2300 Mb | 40,557 | 49 | 1.2 |
| Dicots | *Eucalyptus grandis* | 691 Mb | 36,349 | 40 | 1.1 |
|  | *Populus trichocarpa* | 422.9 Mb | 42,950 | 52 | 1.2 |
|  | *Medicago truncatula* | 390 Mb | 62,319 | 29 | 0.47 |
|  | *Arabidopsis thaliana* | 135 Mb | 27,416 | 25 | 0.91 |
|  | *Aquilegia coerulea* | 306.5 Mb | 30,023 | 22 | 0.73 |

The data obtained from the Phytozome 12 and the ConGenIE.

*Volvox carter*i v2.1, *Chlamydomonas reinhardtii* v5.5, *Marchantia polymorpha* v3.1 (Common liverwort), *Physcomitrella patens* v3.3, *Selaginella moellendorffii* v1.0, *Picea abies* v1.0, *Pinus taeda* v1.0, *Amborella trichopoda* v1.0, *Oryza sativa* v7_JGI (Rice), *Zea mays* (Ensembl-18)*Brachypodium distachyon* Bd21-3 v1.1 (Purple false brome), *Zea mays* , *Eucalyptus grandis* v2.0 (Rose gum), *Populus trichocarp*a v3.1 (Poplar), *Medicago truncatula* Mt4.0v1 (Barrel medic), *Arabidopsis thaliana* TAIR10, *Aquilegia coerulea* v3.1,
