# Supplementary material for: Comprehensive genomic analysis of the DUF4228 gene family in land plants and expression profiling of ATDUF4228 under abiotic stresses
Source: BMC Genomics. 2020 Jan 3;21:12. doi: 10.1186/s12864-019-6389-3 (PMC6942412; doi:10.1186/s12864-019-6389-3)
Supplement: Supplementary file 2 — Additional file 2: Table S2. List of DUF4228 genes included in this study and the features of their encoded proteins. Those DUF4228 proteins with a domain coverage of less than 70% are labelled in yellow. The genes are marked in red because some amino acid residues in their protein sequence are represented by “X”. [file 12864_2019_6389_MOESM2_ESM.docx]

**Table S2** List of *DUF4228* genes included in the study and their encoded protein features. The DUF4228 proteins with a domain coverage of less than 70% are labeled in yellow. The genes are marked in red because some amino acid residues in their protein sequence are represented by “X”.

| Locus ID | No. of a.a | Mass (Da) | pI | Subcellular  localization |
| --- | --- | --- | --- | --- |
| ***Marchantia polymorpha*** |  |  |  |  |
| *Mapoly0030s0105.1.p* | 175 | 19471.87 | 6.59 | nucl |
| *Mapoly0138s0029.1.p* | 174 | 20289.12 | 7.77 | nucl |
| *Mapoly0070s0100.1.p* | 254 | 28659.65 | 9.69 | nucl |
| *Mapoly0070s0097.1.p* | 211 | 23080.39 | 9.39 | nucl |
| *Mapoly0192s0002.1.p* | 234 | 24717.58 | 9.87 | extr |
| *Mapoly0212s0004.1.p* | 295 | 32448.14 | 9.48 | nucl |
| *Mapoly0013s0104.1.p* | 228 | 25695.72 | 9.30 | chlo nucl |
| *Mapoly0039s0033.1.p* | 216 | 24185.43 | 9.10 | nucl |
| *Mapoly0131s0033.1.p* | 233 | 25266.93 | 9.35 | chlo |
| *Mapoly0053s0029.1.p* | 309 | 34285.01 | 9.94 | nucl |
| *Mapoly0169s0012.1.p* | 238 | 26742.37 | 9.36 | nucl |
| *Mapoly0007s0287.1.p* | 247 | 26608.02 | 10.29 | nucl |
| *Mapoly0024s0004.1.p* | 192 | 21884.22 | 9.97 | nucl |
| *Mapoly0008s0222.1.p* | 234 | 25816.82 | 10.25 | cyto |
| *Mapoly0171s0007.1.p* | 188 | 20553.10 | 9.96 | mito |
| *Mapoly0049s0110.1.p* |  |  |  |  |
| *Mapoly0013s0103.1.p* |  |  |  |  |
| ***Physcomitrella patens*** |  |  |  |  |
| *Pp3c10_7880V3.1.p* | 198 | 22234.36 | 9.54 | nucl |
| *Pp3c1_38210V3.1.p* | 207 | 23156.68 | 10.08 | cyto |
| *Pp3c12_1920V3.1.p* | 203 | 22672.80 | 10.13 | nucl |
| *Pp3c12_2990V3.1.p* | 327 | 36924.37 | 9.78 | nucl |
| *Pp3c13_5590V3.1.p* | 233 | 26538.96 | 9.31 | nucl |
| *Pp3c13_5660V3.1.p* | 331 | 37100.60 | 9.96 | nucl |
| *Pp3c14_22510V3.1.p* | 197 | 22022.16 | 9.86 | nucl |
| *Pp3c15_2840V3.1.p* | 204 | 22390.68 | 9.59 | nucl |
| *Pp3c2_3800V3.1.p* | 209 | 22965.99 | 8.70 | nucl |
| *Pp3c23_10020V3.1.p* | 212 | 24561.11 | 9.12 | chlo |
| *Pp3c3_2140V3.1.p* | 334 | 37800.31 | 10.18 | nucl |
| *Pp3c3_2160V3.1.p* | 261 | 28910.36 | 9.25 | nucl |
| *Pp3c3_2190V3.1.p* | 248 | 28106.46 | 9.60 | nucl |
| *Pp3c4_21860V3.1.p* | 262 | 29235.51 | 9.84 | nucl |
| *Pp3c4_21870V3.1.p* | 234 | 26444.70 | 9.63 | chlo |
| *Pp3c7_130V3.1.p* | 181 | 20206.21 | 9.58 | nucl |
| *Pp3c7_180V3.1.p* | 182 | 20091.10 | 9.60 | cyto |
| *Pp3c9_2480V3.1.p* | 205 | 22444.61 | 9.69 | nucl |
| *Pp3c24_13210V3.1.p* |  |  |  |  |
| ***Selaginella moellendorffii*** |  |  |  |  |
| *403062* | 165 | 17879.65 | 9.60 | nucl |
| *440323* | 191 | 21143.11 | 9.74 | nucl |
| *407681* | 278 | 29410.92 | 9.82 | extr |
| ***Picea abies*** |  |  |  |  |
| *MA_10001015g0010* | 179 | 20262.91 | 7.08 | nucl |
| *MA_10165933g0010* | 180 | 20352.36 | 8.56 | chlo |
| *MA_10169333g0010* | 170 | 19154.15 | 8.61 | chlo |
| *MA_10238024g0010* | 186 | 20693.49 | 7.63 | chlo |
| *MA_10296634g0010* | 180 | 20223.12 | 6.72 | nucl |
| *MA_10396g0010* | 233 | 26715.57 | 8.47 | nucl |
| *MA_10425793g0010* | 229 | 25327.03 | 9.87 | cyto |
| *MA_10427211g0020* | 196 | 21939.18 | 8.27 | cyto |
| *MA_10427551g0010* | 179 | 20035.82 | 8.17 | nucl |
| *MA_10427662g0010* | 221 | 24422.30 | 9.74 | nucl |
| *MA_10428693g0010* | 203 | 22807.40 | 7.72 | nucl_plas |
| *MA_10430658g0010* | 183 | 20595.39 | 6.88 | nucl |
| *MA_10432844g0010* | 217 |  |  | chlo |
| *MA_10435613g0010* | 226 | 25154.98 | 8.57 | cyto |
| *MA_10436843g0030* | 254 | 20640.86 | 9.48 | chlo |
| *MA_117824g0010* | 201 | 21991.09 | 5.78 | cyto |
| *MA_118095g0010* | 224 | 24908.83 | 9.08 | chlo |
| *MA_118363g0010* | 227 | 25939.84 | 10.76 | nucl |
| *MA_119175g0010* | 187 | 21324.23 | 6.21 | chlo nucl extr: |
| *MA_120628g0010* | 227 | 25517.37 | 8.21 | cyto |
| *MA_121280g0010* | 190 | 21303.29 | 5.15 | mito |
| *MA_13068g0010* | 210 | 23841.76 | 9.30 | chlo |
| *MA_134509g0010* | 187 | 20551.33 | 6.10 | nucl |
| *MA_138974g0010* | 224 | 25142.06 | 9.32 | chlo |
| *MA_140980g0010* | 182 | 20265.21 | 6.75 | nucl |
| *MA_16509g0010* | 216 | 23343.20 | 4.83 | nucl |
| *MA_182245g0010* | 215 | 23962.34 | 8.24 | nucl |
| *MA_193829g0010* | 183 | 20596.66 | 9.49 | mito: chlo_mito |
| *MA_212504g0010* | 214 | 24279.09 | 9.87 | nucl |
| *MA_218141g0010* | 202 | 22057.27 | 9.86 | extr |
| *MA_218732g0010* | 232 | 25777.74 | 9.94 | nucl |
| *MA_21964g0010* | 197 | 22061.29 | 6.82 | chlo |
| *MA_223132g0010* | 227 | 25174.96 | 9.65 | nucl extr |
| *MA_24094g0010* | 204 | 22849.47 | 8.79 | chlo |
| *MA_25590g0010* | 191 | 21530.88 | 9.02 | cyto |
| *MA_258056g0010* | 144 | 16294.15 | 10.67 | chlo |
| *MA_32672g0010* | 225 | 25551.88 | 6.83 | nucl |
| *MA_34792g0010* | 158 | 17758.32 | 7.00 | nucl |
| *MA_353082g0010* | 182 | 20210.17 | 8.76 | Chlo nucl |
| *MA_374419g0010* | 221 | 24824.53 | 8.58 | nucl extr |
| *MA_376602g0010* | 229 | 25815.59 | 9.37 | nucl |
| *MA_38919g0010* | 188 | 20838.12 | 8.91 | chlo |
| *MA_41993g0010* | 230 | 26087.09 | 8.93 | nucl |
| *MA_469837g0010* | 157 | 17321.27 | 8.52 | pero |
| *MA_495117g0010* | 188 | 21070.28 | 7.47 | nucl |
| *MA_531422g0010* | 196 | 21491.45 | 5.18 | chlo |
| *MA_5824616g0010* | 228 | 25274.28 | 8.83 | extr |
| *MA_59045g0010* | 187 | 20752.79 | 8.44 | nucl |
| *MA_593637g0010* | 174 | 19087.91 | 7.66 | nucl |
| *MA_600517g0010* | 218 | 24147.81 | 9.10 | Nucl extr |
| *MA_6324950g0010* | 204 | 22680.18 | 6.59 | nucl |
| *MA_76101g0010* | 223 | 24894.81 | 8.19 | cyto cyto_nucl |
| *MA_82882g0010* | 205 |  |  | chlo |
| *MA_8819g0010* | 226 | 25288.23 | 8.44 | nucl |
| *MA_88642g0010* | 244 | 27311.87 | 9.44 | nucl extr |
| *MA_897390g0010* | 199 | 22489.89 | 6.53 | cyto |
| *MA_9271818g0010* | 194 | 21427.51 | 5.26 | chlo |
| *MA_96850g0010* | 228 | 26292.01 | 9.34 | nucl |
| *MA_10436027g0020* | 197 | 22200.47 | 8.99 | nucl |
| *MA_20252g0010* | 151 | 17168.42 | 7.03 | cyto |
| *MA_248791g0010* | 232 | 25876.24 | 8.89 | nucl |
| *MA_311429g0010* | 183 | 20900.23 | 9.39 | nucl cyto |
| *MA_356354g0010* | 200 | 21809.83 | 9.27 | nucl |
| *MA_396124g0010* | 186 | 21326.16 | 6.20 | nucl |
| *MA_6869016g0010* | 206 | 23323.93 | 8.63 | nucl |
| *MA_853405g0010* | 201 | 22044.00 | 9.47 | nucl |
| *MA_9305572g0010* | 188 | 21073.03 | 9.98 | cyto |
| *MA_10381881g0010* | 197 | 22451.09 | 9.77 | nucl |
| *MA_152921g0010* | 186 | 21098.21 | 8.17 | cyto |
| *MA_9762162g0010* | 203 | 22838.41 | 6.10 | chlo |
| *MA_124602g0010* | 186 | 21030.98 | 9.27 | chlo |
| *MA_126994g0010* | 198 | 22426.93 | 8.68 | nucl cyto |
| *MA_451787g0010* | 171 | 19283.22 | 7.17 | nucl |
| *MA_9747857g0010* | 171 | 19283.22 | 7.17 | nucl |
| *MA_111971g0020* | 207 | 23489.87 | 9.86 | nucl |
| *MA_20174g0010* | 214 | 23866.35 | 10.13 | mito |
| *MA_953514g0010* | 169 | 18707.25 | 9.75 | mito |
| *MA_37203g0010* | 200 | 22452.69 | 10.05 | mito |
| *MA_64313g0010* | 171 | 19595.45 | 8.47 | nucl |
| *MA_10882g0010* | 209 | 23335.62 | 9.77 | mito |
| *MA_491186g0010* | 180 | 20393.50 | 9.99 | nucl |
| *MA_568302g0010* | 187 | 21290.33 | 9.73 | nucl |
| *MA_926091g0010* | 217 | 24495.21 | 10.13 | cyto |
| *MA_168522g0010* | 210 | 23281.14 | 8.67 | extr |
| *MA_203651g0010* | 252 | 28460.28 | 9.88 | nucl |
| *MA_10276523g0010* | 242 | 27910.68 | 9.56 | nucl |
| *MA_269349g0010* | 235 | 26125.09 | 6.00 | nucl |
| *MA_804676g0010* | 233 | 26176.22 | 8.28 | chlo |
| *MA_10436843g0020* | 254 |  |  | nucl |
| *MA_683856g0010* | 282 | 31560.43 | 7.81 | extr |
| *MA_10248626g0010* | 324 | 36026.93 | 6.08 | nucl |
| *MA_101204g0010* |  |  |  |  |
| *MA_10426045g0020* |  |  |  |  |
| *MA_10427270g0010* |  |  |  |  |
| *MA_10427270g0020* |  |  |  |  |
| *MA_10431168g0010* |  |  |  |  |
| *MA_10435480g0010* |  |  |  |  |
| *MA_10436027g0010* |  |  |  |  |
| *MA_112931g0010* |  |  |  |  |
| *MA_120551g0010* |  |  |  |  |
| *MA_12798g0010* |  |  |  |  |
| *MA_1292008g0010* |  |  |  |  |
| *MA_132098g0020* |  |  |  |  |
| *MA_185992g0010* |  |  |  |  |
| *MA_199566g0010* |  |  |  |  |
| *MA_211219g0010* |  |  |  |  |
| *MA_212588g0010* |  |  |  |  |
| *MA_224816g0010* |  |  |  |  |
| *MA_31046g0010* |  |  |  |  |
| *MA_39044g0010* |  |  |  |  |
| *MA_394398g0010* |  |  |  |  |
| *MA_4151961g0010* |  |  |  |  |
| *MA_43105g0010* |  |  |  |  |
| *MA_4360836g0010* |  |  |  |  |
| *MA_454162g0010* |  |  |  |  |
| *MA_4605800g0010* |  |  |  |  |
| *MA_4761105g0010* |  |  |  |  |
| *MA_496283g0010* |  |  |  |  |
| *MA_4985671g0010* |  |  |  |  |
| *MA_536291g0010* |  |  |  |  |
| *MA_6390114g0010* |  |  |  |  |
| *MA_6741703g0010* |  |  |  |  |
| *MA_70309g0010* |  |  |  |  |
| *MA_76415g0010* |  |  |  |  |
| *MA_836238g0010* |  |  |  |  |
| *MA_84389g0010* |  |  |  |  |
| *MA_87298g0010* |  |  |  |  |
| *MA_91700g0010* |  |  |  |  |
| *MA_9945909g0010* |  |  |  |  |
| ***Pinus taeda*** |  |  |  |  |
| *PITA_000094977-RA* | 175 | 19682.59 | 8.63 | chlo |
| *PITA_000052848-RA* | 264 | 29139.32 | 7.66 | nucl |
| *PITA_000032313-RA* | 268 | 28357.67 | 4.72 | extr |
| *PITA_000062616-RA* | 151 | 17343.86 | 6.53 | mito |
| *PITA_000067574-RA* | 133 | 15109.47 | 5.69 | cyto mito |
| *PITA_000040021-RA* | 290 | 31961.46 | 8.53 | nucl |
| *PITA_000070571-RA* | 404 | 44699.32 | 9.57 | nucl |
| *PITA_000054720-RA* | 276 | 30481.93 | 7.99 | mito |
| *PITA_000026600-RA* | 437 | 49297.45 | 6.63 | nucl |
| *PITA_000064821-RA* | 177 | 20360.41 | 8.66 | nucl |
| *PITA_000052545-RA* | 216 | 24019.26 | 5.49 | chlo |
| *PITA_000050394-RA* | 301 | 33164.80 | 7.96 | mito |
| *PITA_000029829-RA* | 260 | 28843.44 | 7.50 | chlo |
| *PITA_000056739-RA* | 266 | 30229.22 | 5.85 | chlo |
| *PITA_000031629-RA* | 383 | 42891.91 | 5.90 | nucl |
| *PITA_000080774-RA* | 213 | 23650.08 | 5.79 | extr |
| *PITA_000051368-RA* | 168 | 19087.73 | 7.11 | nucl |
| *PITA_000047460-RA* | 250 | 28375.07 | 7.58 | chlo |
| *PITA_000025997-RA* | 217 | 23341.19 | 4.89 | nucl |
| *PITA_000085290-RA* | 260 | 29498.25 | 9.48 | nucl |
| *PITA_000063506-RA* | 241 | 27139.87 | 8.90 | nucl |
| *PITA_000031250-RA* | 364 | 40435.11 | 8.70 | mito |
| *PITA_000028766-RA* | 372 | 41529.66 | 9.04 | chlo |
| *PITA_000027867-RA* | 621 | 69074.51 | 9.25 | nucl |
| *PITA_000050584-RA* | 398 | 45318.11 | 8.23 | chlo |
| *PITA_000029124-RA* | 888 | 97612.42 | 8.96 | chlo |
| *PITA_000085886-RA* | 294 | 32188.81 | 9.73 | nucl |
| *PITA_000018195-RA* | 449 | 51180.73 | 9.06 | cyto |
| *PITA_000052174-RA* | 203 | 23519.12 | 10.16 | mito |
| *PITA_000089830-RA* | 322 | 35788.90 | 8.88 | mito |
| *PITA_000052311-RA* | 224 | 25410.29 | 8.90 | cyto |
| *PITA_000033628-RA* | 385 | 43336.62 | 7.55 | mito |
| *PITA_000082515-RA* | 225 | 25730.58 | 8.56 | chlo |
| *PITA_000033731-RA* | 399 | 44603.90 | 7.20 | cyto |
| *PITA_000000664-RA* | 169 | 18674.72 | 9.06 | nucl |
| *PITA_000061052-RA* | 258 | 29104.51 | 7.58 | nucl |
| *PITA_000071673-RA* | 393 | 43687.80 | 6.94 | extr |
| *PITA_000059078-RA* | 160 | 18435.02 | 5.18 | cyto_nucl |
| *PITA_000053371-RA* | 248 | 27307.02 | 9.57 | mito |
| *PITA_000037787-RA* | 165 | 18203.77 | 9.08 | cyto |
| *PITA_000056740-RA* | 191 | 21918.62 | 5.50 | chlo |
| *PITA_000045642-RA* | 157 | 17851.26 | 5.69 | cyto |
| *PITA_000049496-RA* | 202 | 22381.69 | 8.77 | chlo nucl |
| *PITA_000028595-RA* | 206 | 22206.19 | 5.43 | chlo |
| *PITA_000084522-RA* | 409 | 44659.00 | 9.48 | chlo |
| *PITA_000050369-RA* | 400 | 44909.14 | 6.86 | chlo |
| *PITA_000085756-RA* | 152 | 17084.72 | 9.10 | mito |
| *PITA_000063810-RA* | 215 | 24553.28 | 8.09 | nucl |
| *PITA_000061546-RA* | 195 | 22613.09 | 5.59 | cyto |
| *PITA_000052546-RA* | 201 | 22777.63 | 5.09 | extr |
| *PITA_000035905-RA* | 324 | 36141.18 | 8.88 | chlo |
| *PITA_000035904-RA* | 270 | 30213.12 | 8.29 | cyto |
| *PITA_000017438-RA* | 547 | 60048.70 | 9.70 | nucl |
| *PITA_000005619-RA* | 240 | 27024.35 | 8.15 | chlo |
| *PITA_000088421-RA* | 205 | 23084.65 | 8.58 | nucl cyto |
| *PITA_000035175-RA* | 210 | 23321.94 | 9.40 | chlo |
| *PITA_000041260-RA* |  |  |  |  |
| *PITA_000041260-RA* |  |  |  |  |
| *PITA_000049284-RA* |  |  |  |  |
| *PITA_000081096-RA* |  |  |  |  |
| *PITA_000091437-RA* |  |  |  |  |
| *PITA_000069400-RA* |  |  |  |  |
| *>PITA_000055698-RA* |  |  |  |  |
| *PITA_000063811-RA* |  |  |  |  |
| *PITA_000066739-RA* |  |  |  |  |
| *PITA_000017439-RA* |  |  |  |  |
| *PITA_000009713-RA* |  |  |  |  |
| *PITA_000092169-RA* |  |  |  |  |
| *PITA_000081971-RA* |  |  |  |  |
| *PITA_000082262-RA* |  |  |  |  |
| *PITA_000075762-RA* |  |  |  |  |
| *PITA_000071673-RA* |  |  |  |  |
| *PITA_000045101-RA* |  |  |  |  |
| *PITA_000032905-RA* |  |  |  |  |
| *PITA_000064447-RA* |  |  |  |  |
| *PITA_000091928-RA* |  |  |  |  |
| *PITA_000065488-RA* |  |  |  |  |
| *PITA_000057868-RA* |  |  |  |  |
| *PITA_000088137-RA* |  |  |  |  |
| *PITA_000088137-RA* |  |  |  |  |
| *PITA_000026069-RA* |  |  |  |  |
| *PITA_000026069-RA* |  |  |  |  |
| *PITA_000026067-RA* |  |  |  |  |
| *PITA_000026066-RA* |  |  |  |  |
| *PITA_000031629-RA* |  |  |  |  |
| *PITA_000056739-RA* |  |  |  |  |
| *PITA_000038455-RA* |  |  |  |  |
| *PITA_000050394-RA* |  |  |  |  |
| *PITA_000057240-RA* |  |  |  |  |
| *PITA_000083524-RA* |  |  |  |  |
| *PITA_000088129-RA* |  |  |  |  |
| *PITA_000088129-RA* |  |  |  |  |
| *PITA_000083021-RA* |  |  |  |  |
| *PITA_000033342-RA* |  |  |  |  |
| *PITA_000057146-RA* |  |  |  |  |
| *PITA_000061741-RA* |  |  |  |  |
| *PITA_000084522-RA* |  |  |  |  |
| *PITA_000074420-RA* |  |  |  |  |
| *PITA_000081493-RA* |  |  |  |  |
| *PITA_000070349-RA* |  |  |  |  |
| *PITA_000078805-RA* |  |  |  |  |
| *PITA_000087999-RA* |  |  |  |  |
| *PITA_000029124-RA* |  |  |  |  |
| *PITA_000029124-RA* |  |  |  |  |
| *PITA_000065306-RA* |  |  |  |  |
| *PITA_000080078-RA* |  |  |  |  |
| *PITA_000083856-RA* |  |  |  |  |
| *PITA_000042805-RA* |  |  |  |  |
| *PITA_000056848-RA* |  |  |  |  |
| *PITA_000036806-RA* |  |  |  |  |
| *PITA_000092165-RA* |  |  |  |  |
| *PITA_000067407-RA* |  |  |  |  |
| *PITA_000074952-RA* |  |  |  |  |
| *PITA_000070183-RA* |  |  |  |  |
| *PITA_000054720-RA* |  |  |  |  |
| *PITA_000089830-RA* |  |  |  |  |
| *PITA_000004135-RA* |  |  |  |  |
| *PITA_000026600-RA* |  |  |  |  |
| *PITA_000029829-RA* |  |  |  |  |
| *PITA_000061708-RA* |  |  |  |  |
| *PITA_000024142-RA* |  |  |  |  |
| *PITA_000028766-RA* |  |  |  |  |
| *PITA_000018195-RA* |  |  |  |  |
| ***Amborella trichopoda*** |  |  |  |  |
| *evm_27.model.AmTr_v1.0_scaffold00022.22* | 187 | 20543.75 | 9.74 | nucl |
| *evm_27.model.AmTr_v1.0_scaffold00065.106* | 159 | 17738.20 | 9.94 | nucl |
| *evm_27.model.AmTr_v1.0_scaffold00105.76* | 178 | 20175.39 | 5.98 | chlo |
| *evm_27.model.AmTr_v1.0_scaffold00105.75* | 188 | 20976.04 | 8.37 | extr |
| *evm_27.model.AmTr_v1.0_scaffold00002.14* | 219 | 24302.04 | 9.86 | nucl |
| *evm_27.model.AmTr_v1.0_scaffold00113.6* | 203 | 22189.04 | 5.35 | nucl |
| *evm_27.model.AmTr_v1.0_scaffold00113.19* | 161 | 17821.24 | 5.15 | chlo |
| *evm_27.model.AmTr_v1.0_scaffold00021.24* | 151 | 17287.34 | 9.60 | cyto |
| *evm_27.model.AmTr_v1.0_scaffold00021.288* | 169 | 19038.02 | 5.34 | cyto extr |
| *evm_27.model.AmTr_v1.0_scaffold00021.221* | 191 | 21010.70 | 4.74 | nucl |
| *evm_27.model.AmTr_v1.0_scaffold00025.205* | 178 | 19257.34 | 10.22 | nucl |
| *evm_27.model.AmTr_v1.0_scaffold00077.175* | 143 | 16213.64 | 8.94 | nucl |
| *evm_27.model.AmTr_v1.0_scaffold00064.94* | 204 | 22981.56 | 8.85 | chlo |
| *evm_27.model.AmTr_v1.0_scaffold00063.42* | 194 | 21775.25 | 9.20 | chlo |
| *evm_27.model.AmTr_v1.0_scaffold00016.365* | 267 | 28848.45 | 9.11 | nucl |
| *evm_27.model.AmTr_v1.0_scaffold00016.280* | 245 | 28039.78 | 10.04 | nucl |
| *evm_27.model.AmTr_v1.0_scaffold00010.33* | 198 | 21926.41 | 9.64 | vacu |
| *evm_27.model.AmTr_v1.0_scaffold00023.48* | 171 | 18874.00 | 9.42 | extr |
| *evm_27.model.AmTr_v1.0_scaffold00057.20* | 155 | 17091.48 | 6.43 | mito |
| *evm_27.model.AmTr_v1.0_scaffold00057.22* | 191 | 21620.16 | 9.62 | chlo |
| *evm_27.model.AmTr_v1.0_scaffold00049.218* | 195 | 22656.28 | 9.61 | nucl |
| *evm_27.model.AmTr_v1.0_scaffold00163.30* |  |  |  |  |
| *evm_27.model.AmTr_v1.0_scaffold00163.27* |  |  |  |  |
| *evm_27.model.AmTr_v1.0_scaffold00059.176* |  |  |  |  |
| ***Oryza sativa*** |  |  |  |  |
| *LOC_Os01g03080.1* | 201 | 20876.52 | 6.15 | chlo |
| *LOC_Os01g07150.1* | 170 | 18019.37 | 9.33 | extr |
| *LOC_Os01g19880.1* | 164 | 17297.79 | 8.81 | chlo |
| *LOC_Os01g49210.1* | 188 | 19425.17 | 6.92 | chlo |
| *LOC_Os01g55370.1* | 195 | 21262.65 | 9.67 | cyto |
| *LOC_Os01g72360.1* | 218 | 23152.80 | 8.80 | chlo |
| *LOC_Os02g09970.1* | 209 | 22078.16 | 7.72 | chlo |
| *LOC_Os02g09980.1* | 200 | 21236.37 | 10.08 | chlo |
| *LOC_Os02g09990.1* | 192 | 20321.45 | 9.93 | chlo |
| *LOC_Os02g49960.1* | 246 | 26090.38 | 11.13 | chlo |
| *LOC_Os02g50810.1* | 188 | 20720.97 | 6.61 | chlo nucl cyto |
| *LOC_Os03g10320.1* | 215 | 22984.97 | 7.03 | nucl, cyto |
| *LOC_Os03g18660.1* | 178 | 19245.17 | 9.56 | cyto |
| *LOC_Os03g19260.1* | 209 | 22374.36 | 9.97 | nucl |
| *LOC_Os03g24100.1* | 264 | 28808.02 | 9.55 | cyto |
| *LOC_Os03g52940.1* | 170 | 17017.08 | 7.70 | chlo |
| *LOC_Os03g55880.1* | 140 | 14600.61 | 7.91 | nucl |
| *LOC_Os03g62770.1* | 294 | 32580.48 | 8.80 | chlo |
| *LOC_Os05g07240.1* | 170 | 17969.50 | 9.67 | extr |
| *LOC_Os05g31970.1* | 223 | 22930.27 | 7.62 | nucl extr |
| *LOC_Os05g39710.1* | 171 | 18097.78 | 9.53 | extr |
| *LOC_Os05g47960.1* | 175 | 18612.33 | 5.75 | chlo |
| *LOC_Os06g12960.1* | 189 | 20688.30 | 10.29 | cyto |
| *LOC_Os06g14780.1* | 294 | 31969.90 | 11.20 | chlo |
| *LOC_Os06g37190.1* | 211 | 22391.54 | 10.76 | cyto |
| *LOC_Os06g48680.1* | 193 | 19997.46 | 8.34 | mito |
| *LOC_Os07g03120.1* | 205 | 21203.01 | 5.45 | nucl |
| *LOC_Os07g46510.1* | 233 | 24849.89 | 10.30 | chlo |
| *LOC_Os08g32520.1* | 189 | 20058.52 | 10.00 | chlo |
| *LOC_Os09g21380.1* | 208 | 21657.12 | 9.51 | nucl |
| *LOC_Os09g23780.1* | 246 | 25953.14 | 10.70 | chlo |
| *LOC_Os09g32000.1* | 201 | 20327.85 | 10.21 | chlo |
| *LOC_Os10g21130.1* | 218 | 22203.72 | 7.87 | nucl |
| *LOC_Os11g33050.1* | 280 | 30020.19 | 9.13 | chlo |
| *LOC_Os01g13670.1* |  |  |  |  |
| *LOC_Os06g14770.1* |  |  |  |  |
| *LOC_Os06g41940.1* |  |  |  |  |
| *LOC_Os08g33210.1* |  |  |  |  |
| *LOC_Os09g31990.1* |  |  |  |  |
| ***Brachypodium distachyon*** |  |  |  |  |
| *Bradi1g01890.1.p* | 314 | 34134.21 | 7.62 | chlo |
| *Bradi1g07420.1.p* | 185 | 19223.70 | 6.37 | chlo |
| *Bradi1g09220.2.p* | 244 | 24146.08 | 8.86 | chlo |
| *Bradi1g19020.1.p* | 228 | 24602.99 | 11.13 | chlo |
| *Bradi1g34120.1.p* | 187 | 19288.60 | 8.62 | Chlo nucl |
| *Bradi1g35690.1.p* | 191 | 19878.44 | 10.22 | mito |
| *Bradi1g37579.1.p* | 219 | 23095.03 | 10.65 | nucl |
| *Bradi1g44040.1.p* | 292 | 31644.27 | 10.68 | chlo |
| *Bradi1g44870.1.p* | 182 | 20211.51 | 9.90 | cyto |
| *Bradi1g55825.1.p* | 185 | 19411.21 | 6.40 | nucl |
| *Bradi1g55830.1.p* | 178 | 18342.15 | 9.46 | nucl |
| *Bradi1g62080.2.p* | 231 | 24241.05 | 9.87 | chlo |
| *Bradi1g64620.1.p* | 193 | 21150.14 | 9.51 | cyto |
| *Bradi1g65090.1.p* | 173 | 18558.46 | 9.71 | Chlo plas |
| *Bradi1g71210.1.p* | 209 | 22194.24 | 7.80 | cyto |
| *Bradi2g04050.2.p* | 221 | 23673.65 | 9.54 | chlo vacu |
| *Bradi2g11658.1.p* | 160 | 17282.96 | 10.09 | chlo |
| *Bradi2g17310.2.p* | 167 | 17424.91 | 10.75 | mito |
| *Bradi2g22450.1.p* | 178 | 18983.74 | 10.13 | extr |
| *Bradi2g26817.1.p* | 214 | 22288.77 | 9.30 | nucl extr |
| *Bradi2g34540.1.p* | 162 | 16993.63 | 9.50 | extr |
| *Bradi2g46810.1.p* | 187 | 19430.30 | 6.76 | chlo |
| *Bradi2g50400.1.p* | 190 | 20593.85 | 9.68 | extr |
| *Bradi2g53770.1.p* | 166 | 17889.59 | 9.65 | chlo |
| *Bradi2g60980.1.p* | 254 | 26522.29 | 9.13 | chlo |
| *Bradi2g61030.1.p* | 255 | 26454.11 | 9.13 | chlo |
| *Bradi3g06800.1.p* | 200 | 21020.22 | 9.47 | chlo |
| *Bradi3g36020.1.p* | 189 | 20683.42 | 10.18 | nucl |
| *Bradi3g36407.1.p* | 260 | 28046.53 | 10.01 | chlo |
| *Bradi3g59607.2.p* | 193 | 21060.37 | 7.95 | cyto |
| *Bradi4g17060.1.p* | 291 | 31735.00 | 7.61 | chlo |
| *Bradi4g29450.1.p* | 194 | 20483.92 | 9.79 | nucl |
| *Bradi4g33950.1.p* | 199 | 20342.97 | 10.16 | chlo |
| *Bradi4g33970.1.p* | 192 | 19688.53 | 9.95 | chlo |
| *Bradi4g33975.1.p* | 170 | 17723.93 | 11.24 | chlo |
| *Bradi4g29852.1.p* |  |  |  |  |
| *Bradi3g06790.1.p* |  |  |  |  |
| *Bradi3g56952.1.p* |  |  |  |  |
| ***Zea mays*** |  |  |  |  |
| *AC210892.4_FGP006* | 197 | 20524.98 | 6.22 | nucl |
| *AC234157.1_FGP003* | 182 | 18835.45 | 5.57 | cyto |
| *GRMZM2G001200_P01* | 314 | 33929.35 | 12.02 | chlo |
| *GRMZM2G003821_P01* | 200 | 20867.76 | 9.40 | chlo |
| *GRMZM2G010016_P01* | 144 | 15179.25 | 9.13 | chlo |
| *GRMZM2G023683_P01* | 211 | 22225.67 | 8.75 | chlo |
| *GRMZM2G026783_P01* | 179 | 18802.44 | 9.21 | vacu |
| *GRMZM2G031991_P01* | 173 | 19147.40 | 8.82 | cyto |
| *GRMZM2G033175_P01* | 286 | 30667.18 | 11.18 | chlo |
| *GRMZM2G036282_P01* | 169 | 18683.91 | 8.85 | chlo |
| *GRMZM2G043396_P01* | 174 | 18660.20 | 10.19 | Chlo extr |
| *GRMZM2G052467_P01* | 186 | 19125.72 | 7.67 | nucl |
| *GRMZM2G055809_P03* | 184 | 19041.75 | 10.01 | mito |
| *GRMZM2G057506_P02* | 325 | 34343.23 | 9.99 | chlo |
| *GRMZM2G061103_P02* | 263 | 28874.24 | 10.20 | chlo |
| *GRMZM2G073548_P01* | 249 | 25597.06 | 9.22 | chlo |
| *GRMZM2G082504_P01* | 214 | 22578.85 | 9.54 | chlo |
| *GRMZM2G092525_P01* | 256 | 26941.25 | 10.52 | chlo |
| *GRMZM2G095798_P01* | 166 | 16464.73 | 8.77 | nucl |
| *GRMZM2G097342_P01* | 186 | 19503.96 | 10.13 | chlo |
| *GRMZM2G099413_P01* | 205 | 21033.90 | 9.30 | chlo |
| *GRMZM2G108200_P01* | 212 | 22886.04 | 10.92 | nucl |
| *GRMZM2G133095_P01* | 211 | 22177.65 | 9.01 | chlo |
| *GRMZM2G140674_P01* | 217 | 22758.63 | 9.09 | nucl |
| *GRMZM2G145654_P01* | 194 | 19992.76 | 8.47 | chlo cyto |
| *GRMZM2G162305_P01* | 198 | 21184.60 | 9.66 | extr |
| *GRMZM2G169953_P01* | 176 | 18859.60 | 9.91 | Extr vacu: |
| *GRMZM2G171051_P01* | 252 | 26832.13 | 11.21 | chlo |
| *GRMZM2G173377_P01* | 269 | 28415.67 | 9.68 | chlo |
| *GRMZM2G181540_P01* | 209 | 22381.03 | 6.39 | nucl |
| *GRMZM2G316148_P01* | 300 | 33096.40 | 9.48 | chlo |
| *GRMZM2G323387_P01* | 177 | 18440.18 | 8.89 | chlo |
| *GRMZM2G335604_P01* | 240 | 25071.97 | 9.98 | chlo |
| *GRMZM2G350773_P01* | 240 | 24972.53 | 10.45 | chlo |
| *GRMZM2G354190_P01* | 209 | 21542.29 | 10.30 | chlo |
| *GRMZM2G365240_P01* | 161 | 17654.31 | 9.77 | chlo |
| *GRMZM2G379575_P01* | 193 | 20863.61 | 10.61 | nucl |
| *GRMZM2G381838_P01* | 314 | 34266.76 | 7.03 | chlo |
| *GRMZM2G382711_P01* | 263 | 28003.20 | 11.02 | chlo |
| *GRMZM2G389070_P01* | 147 | 16050.65 | 9.42 | cyto |
| *GRMZM2G399218_P01* | 170 | 18158.66 | 9.90 | cyto extr vacu |
| *GRMZM2G404126_P01* | 205 | 21254.33 | 9.43 | chlo |
| *GRMZM2G406155_P01* | 215 | 22769.19 | 9.25 | extr |
| *GRMZM2G432679_P01* | 202 | 21146.91 | 9.30 | chlo |
| *GRMZM2G451769_P01* | 201 | 21768.49 | 9.74 | chlo |
| *GRMZM2G454056_P01* | 156 | 16823.18 | 9.68 | cyto |
| *GRMZM2G474088_P01* | 181 | 19755.98 | 9.73 | cyto |
| *GRMZM2G475971_P01* | 214 | 22912.03 | 9.23 | nucl |
| *AC198597.4_FGP001* |  |  |  |  |
| *AC198597.4_FGP001* |  |  |  |  |
| *GRMZM2G051855_P01* |  |  |  |  |
| *GRMZM2G056815_P01* |  |  |  |  |
| *GRMZM2G073303_P01* |  |  |  |  |
| *GRMZM2G089357_P01* |  |  |  |  |
| *GRMZM2G456132_P01* |  |  |  |  |
| ***Eucalyptus grandis*** |  |  |  |  |
| *Eucgr.B00346.1.p* | 206 | 22418.65 | 9.79 | chlo |
| *Eucgr.B00885.1.p* | 163 | 18393.24 | 8.37 | chlo |
| *Eucgr.B00987.1.p* | 163 | 18379.21 | 8.37 | chlo |
| *Eucgr.B02556.1.p* | 179 | 19470.36 | 9.78 | nucl |
| *Eucgr.B03286.1.p* | 216 | 24280.17 | 9.66 | chlo |
| *Eucgr.B03364.1.p* | 193 | 21359.54 | 8.66 | nucl |
| *Eucgr.B03728.1.p* | 216 | 23917.34 | 8.25 | chlo |
| *Eucgr.B03729.1.p* | 216 | 24090.83 | 8.76 | chlo |
| *Eucgr.B04008.1.p* | 151 | 16860.48 | 9.79 | extr |
| *Eucgr.C00123.1.p* | 205 | 22082.09 | 10.02 | mito |
| *Eucgr.C02224.1.p* | 213 | 23066.26 | 9.64 | chlo |
| *Eucgr.D00486.1.p* | 166 | 18399.43 | 9.87 | extr |
| *Eucgr.E00016.1.p* | 174 | 18813.32 | 6.05 | nucl |
| *Eucgr.E00885.1.p* | 207 | 22485.51 | 5.37 | chlo |
| *Eucgr.E01054.1.p* | 210 | 23026.06 | 5.84 | nucl |
| *Eucgr.E01061.1.p* | 209 | 22850.74 | 5.37 | nucl |
| *Eucgr.F01951.1.p* | 206 | 22754.00 | 9.79 | chlo |
| *Eucgr.F02810.1.p* | 176 | 18705.26 | 6.17 | chlo |
| *Eucgr.F02820.1.p* | 228 | 25648.65 | 9.87 | nucl |
| *Eucgr.F03622.1.p* | 268 | 30151.32 | 9.91 | nucl |
| *Eucgr.G00481.1.p* | 162 | 17511.81 | 8.35 | nucl |
| *Eucgr.G00484.1.p* | 162 | 17591.85 | 7.00 | nucl |
| *Eucgr.G00485.1.p* | 161 | 17922.61 | 6.99 | nucl |
| *Eucgr.G00487.1.p* | 162 | 17834.51 | 7.00 | nucl |
| *Eucgr.G00488.1.p* | 162 | 18002.75 | 8.54 | mito |
| *Eucgr.G00489.1.p* | 122 | 13372.48 | 6.16 | nucl |
| *Eucgr.G01546.1.p* | 131 | 14466.80 | 7.70 | nucl |
| *Eucgr.G01889.1.p* | 161 | 17930.67 | 7.65 | nucl |
| *Eucgr.G02193.1.p* | 237 | 25324.04 | 9.99 | chlo |
| *Eucgr.H01053.1.p* | 166 | 17945.70 | 8.69 | cyto |
| *Eucgr.H01054.1.p* | 194 | 21172.09 | 6.97 | nucl |
| *Eucgr.H01353.1.p* | 482 | 54162.98 | 9.17 | nucl |
| *Eucgr.H01932.1.p* | 165 | 17364.86 | 7.59 | cyto |
| *Eucgr.H02314.1.p* | 182 | 19494.82 | 6.60 | cyto |
| *Eucgr.I01235.1.p* | 188 | 20635.43 | 8.87 | chlo |
| *Eucgr.J00699.1.p* | 208 | 22784.98 | 6.21 | cyto |
| *Eucgr.J01660.1.p* | 304 | 33909.88 | 9.66 | nucl |
| *Eucgr.K00425.1.p* | 167 | 17810.47 | 9.80 | chlo |
| *Eucgr.K02033.1.p* | 178 | 19700.47 | 6.01 | nucl |
| *Eucgr.K03540.1.p* | 209 | 23908.73 | 10.08 | mito |
| *Eucgr.E04066.1.p* |  |  |  |  |
| *Eucgr.E04065.1.p* |  |  |  |  |
| *Eucgr.E04063.1.p* |  |  |  |  |
| *Eucgr.E04062.1.p* |  |  |  |  |
| *Eucgr.B02897.1.p* |  |  |  |  |
| *Eucgr.I01029.1.p* |  |  |  |  |
| *Eucgr.G00482.1.p* |  |  |  |  |
| *Eucgr.G00483.1.p* |  |  |  |  |
| ***Populus trichocarpa*** |  |  |  |  |
| *Potri.001G070700.1* | 267 | 30302.96 | 9.53 | nucl extr |
| *Potri.001G089000.1* | 200 | 21951.29 | 5.96 | nucl |
| *Potri.001G276400.1* | 207 | 23039.34 | 7.67 | chlo |
| *Potri.001G448500.1* | 221 | 23988.87 | 6.52 | nucl |
| *Potri.002G002100.1* | 214 | 23759.90 | 9.82 | nucl |
| *Potri.002G178900.1* | 202 | 22476.36 | 6.97 | nucl |
| *Potri.002G196900.1* | 464 | 22342.68 | 9.00 | nucl |
| *Potri.002G217100.1* | 248 | 27403.09 | 10.49 | nucl |
| *Potri.002G242400.1* | 322 | 37062.01 | 9.75 | nucl |
| *Potri.003G160000.1* | 268 | 30336.94 | 9.68 | nucl |
| *Potri.003G179600.1* | 210 | 23784.98 | 8.80 | cyto |
| *Potri.004G057500.1* | 178 | 19780.60 | 9.45 | chlo cyto extr |
| *Potri.004G088200.1* | 216 | 24086.48 | 9.17 | cyto |
| *Potri.004G094000.1* | 178 | 19781.91 | 9.30 | chlo |
| *Potri.004G149600.1* | 176 | 19463.99 | 5.78 | chlo nucl |
| *Potri.005G259100.1* | 211 | 23528.80 | 9.72 | nucl |
| *Potri.006G018200.1* | 150 | 16906.68 | 9.89 | chlo |
| *Potri.006G076500.1* | 174 | 19415.99 | 8.22 | chlo |
| *Potri.006G105500.1* | 167 | 18121.93 | 8.65 | chlo |
| *Potri.006G105600.1* | 167 | 18191.04 | 8.88 | chlo |
| *Potri.006G213100.1* | 164 | 18316.08 | 7.02 | chlo |
| *Potri.007G005600.1* | 184 | 19575.15 | 9.27 | cyto |
| *Potri.007G033400.1* | 154 | 16548.96 | 6.82 | chlo |
| *Potri.008G127200.1* | 179 | 19833.67 | 8.86 | chlo |
| *Potri.008G128700.1* | 215 | 23848.67 | 9.73 | nucl |
| *Potri.008G146500.1* | 223 | 25210.91 | 9.32 | nucl |
| *Potri.008G186700.1* | 203 | 22809.41 | 9.49 | chlo |
| *Potri.009G071100.1* | 207 | 23208.60 | 9.10 | chlo |
| *Potri.010G095400.1* | 180 | 20328.32 | 9.86 | nucl |
| *Potri.010G113800.1* | 207 | 23023.85 | 9.84 | nucl |
| *Potri.010G116000.1* | 178 | 19720.61 | 8.38 | chlo |
| *Potri.011G066500.1* | 179 | 19948.90 | 9.76 | extr |
| *Potri.011G153000.1* | 209 | 22738.58 | 5.94 | extr |
| *Potri.011G153100.1* | 209 | 22738.58 | 5.94 | extr |
| *Potri.012G022300.1* | 168 | 18641.58 | 6.43 | mito |
| *Potri.012G045600.1* | 163 | 18278.99 | 6.73 | extr |
| *Potri.012G077300.1* | 186 | 20502.28 | 9.82 | nucl |
| *Potri.013G153100.1* | 156 | 17280.92 | 9.62 | extr |
| *Potri.013G160200.1* | 166 | 18232.69 | 8.31 | nucl |
| *Potri.014G104900.1* | 201 | 22312.97 | 5.96 | nucl |
| *Potri.014G122100.1* | 194 | 22075.65 | 9.11 | chlo |
| *Potri.014G170000.1* | 238 | 26778.61 | 10.49 | nucl |
| *Potri.015G005700.1* | 168 | 18493.54 | 7.74 | chlo |
| *Potri.015G036800.1* | 162 | 18196.03 | 8.33 | chlo |
| *Potri.015G072500.1* | 124 | 14031.82 | 9.01 | cyto |
| *Potri.016G079700.1* | 159 | 17859.77 | 6.90 | cyto |
| *Potri.016G128800.1* | 171 | 18714.73 | 7.54 | chlo |
| *Potri.017G121200.1* | 178 | 19785.99 | 9.23 | nucl |
| *Potri.017G128800.1* | 216 | 24362.88 | 9.38 | cyto |
| *Potri.018G143700.1* | 172 | 19151.80 | 9.27 | chlo |
| *Potri.019G123900.1* | 166 | 18347.17 | 9.76 | chlo cyto vacu |
| *Potri.T141900.1* | 216 | 23888.58 | 6.20 | chlo |
| *Potri.004G149800.1* |  |  |  |  |
| *Potri.005G130200.1* |  |  |  |  |
| *Potri.009G110600.1* |  |  |  |  |
| *Potri.009G110700.1* |  |  |  |  |
| *Potri.010G046200.1* |  |  |  |  |
| *Potri.014G005800.1* |  |  |  |  |
| *Potri.014G009100.1* |  |  |  |  |
| *Potri.018G030600.1* |  |  |  |  |
| ***Medicago truncatula*** |  |  |  |  |
| *Medtr1g030740.1* | 211 | 23669.63 | 9.71 | nucl |
| *Medtr1g087230.1* | 192 | 22092.44 | 6.66 | cyto |
| *Medtr1g099630.1* | 203 | 22688.66 | 6.38 | Nucl cysk |
| *Medtr1g108640.1* | 173 | 19392.25 | 8.46 | chlo |
| *Medtr2g016150.1* | 186 | 20812.77 | 9.76 | chlo |
| *Medtr2g037760.1* | 184 | 20316.12 | 9.59 | nucl |
| *Medtr2g104800.1* | 246 | 27699.34 | 9.79 | nucl |
| *Medtr2g105800.1* | 156 | 17653.68 | 6.83 | nucl |
| *Medtr3g031140.1* | 170 | 18578.05 | 9.08 | extr |
| *Medtr3g045790.1* | 225 | 25346.21 | 8.57 | chlo |
| *Medtr3g067730.1* | 217 | 23932.66 | 9.93 | nucl |
| *Medtr3g092470.1* | 141 | 15572.97 | 6.90 | cyto |
| *Medtr4g085100.1* | 210 | 23480.39 | 9.94 | nucl |
| *Medtr4g097220.1* | 168 | 18615.33 | 9.95 | nucl |
| *Medtr4g106790.1* | 187 | 21182.74 | 9.49 | nucl |
| *Medtr4g127530.1* | 185 | 20640.71 | 8.98 | chlo |
| *Medtr5g018720.1* | 161 | 17441.78 | 8.75 | chlo |
| *Medtr5g035430.1* | 173 | 19237.99 | 9.71 | chlo |
| *Medtr5g074290.1* | 160 | 18227.93 | 9.24 | extr |
| *Medtr5g079610.1* | 211 | 23597.26 | 9.62 | nucl |
| *Medtr5g089820.1* | 209 | 23676.42 | 6.95 | chlo |
| *Medtr6g052290.1* | 248 | 26808.41 | 8.44 | nucl |
| *Medtr7g109410.1* | 197 | 22552.25 | 8.94 | pero |
| *Medtr7g109420.1* | 157 | 17416.14 | 9.35 | extr |
| *Medtr8g017100.1* | 202 | 22599.20 | 5.97 | extr |
| *Medtr8g063200.1* | 173 | 19596.60 | 9.37 | chlo |
| *Medtr8g078790.1* | 171 | 18478.24 | 6.72 | chlo |
| *Medtr8g099655.1* | 228 | 24906.17 | 6.90 | nucl |
| *Medtr8g107420.1* | 166 | 19019.90 | 6.91 | cyto |
| *Medtr3g078300.2* |  |  |  |  |
| *Medtr4g068850.1* |  |  |  |  |
| *Medtr5g018750.1* |  |  |  |  |
| *Medtr7g057900.1* |  |  |  |  |
| ***Arabidopsis thaliana*** |  |  |  |  |
| *AT1G06980* | 169 | 18942.78 | 9.28 | cyto, vacu |
| *AT1G10530* | 166 | 18811.84 | 10.0 | chlo |
| *AT1G18290* | 176 | 19834.65 | 8.59 | chlo |
| *AT1G21010* | 210 | 22975.17 | 10.04 | nucl |
| *AT1G28190* | 266 | 30336.38 | 9.97 | chlo |
| *AT1G29195* | 193 | 21645.75 | 8.95 | extr |
| *AT1G60010* | 173 | 19603.39 | 9.71 | nucl |
| *AT1G64700* | 203 | 22683.66 | 7.82 | cyto_nucl |
| *AT1G71015* | 195 | 22255.78 | 10.00 | nucl |
| *AT1G76600* | 216 | 23796.86 | 9.93 | nucl |
| *AT2G23690* | 163 | 17648.32 | 9.28 | nucl |
| *AT2G30230* | 177 | 19841.91 | 8.92 | extr |
| *AT3G03280* | 166 | 18244.90 | 6.51 | cyto |
| *AT3G10120* | 173 | 20153.28 | 7.10 | nucl , cyto |
| *AT3G50800* | 152 | 16278.41 | 6.59 | chlo |
| *AT3G61920* | 187 | 19938.67 | 5.83 | nucl |
| *AT4G02090* | 202 | 22316.56 | 9.49 | chlo |
| *AT4G37240* | 168 | 18027.69 | 7.50 | chlo |
| *AT5G03890* | 179 | 20389.16 | 5.66 | cyto |
| *AT5G12340* | 220 | 25105.91 | 8.46 | chlo |
| *AT5G17350* | 183 | 19658.46 | 8.97 | chlo |
| *AT5G50090* | 159 | 17440.25 | 9.70 | nucl |
| *AT5G62900* | 161 | 17630.14 | 9.77 | nucl |
| *AT5G66580* | 156 | 16631.74 | 6.58 | Chlo |
| *AT5G67620* | 182 | 19706.47 | 9.42 | cyto |
| *AT5G37840* |  |  |  |  |
| *AT2G01340* |  |  |  |  |
| *AT1G66480* |  |  |  |  |
| ***Aquilegia coerulea*** |  |  |  |  |
| *Aqcoe0278s0002.1.p* | 165 | 18151.84 | 9.15 | chlo |
| *Aqcoe1G148100.1.p* | 156 | 17564.30 | 9.40 | cyto extr |
| *Aqcoe1G225300.1.p* | 169 | 19155.12 | 9.28 | extr |
| *Aqcoe1G444800.1.p* | 154 | 17184.05 | 8.72 | cyto |
| *Aqcoe2G039700.1.p* | 206 | 22723.45 | 8.59 | chlo |
| *Aqcoe2G081100.1.p* | 162 | 17769.40 | 9.68 | cyto vacu |
| *Aqcoe2G202100.1.p* | 191 | 21737.69 | 9.76 | cyto |
| *Aqcoe2G202200.1.p* | 159 | 17809.36 | 5.16 | chlo |
| *Aqcoe3G003100.1.p* | 265 | 29716.94 | 9.68 | nucl |
| *Aqcoe3G109200.1.p* | 203 | 23067.29 | 9.97 | nucl |
| *Aqcoe3G207500.1.p* | 269 | 30403.37 | 9.82 | chlo |
| *Aqcoe3G303700.1.p* | 180 | 20224.05 | 9.84 | nucl |
| *Aqcoe4G043100.1.p* | 160 | 17929.93 | 9.93 | extr |
| *Aqcoe5G047100.1.p* | 162 | 18013.86 | 6.95 | pero |
| *Aqcoe5G109600.1.p* | 191 | 21347.43 | 9.93 | nucl cyto |
| *Aqcoe5G202900.1.p* | 175 | 18593.13 | 7.60 | chlo |
| *Aqcoe5G400600.1.p* | 205 | 23877.85 | 9.77 | mito |
| *Aqcoe6G030200.1.p* | 223 | 24748.59 | 10.31 | mito |
| *Aqcoe6G104900.1.p* | 176 | 18441.98 | 6.70 | chlo |
| *Aqcoe6G273800.1.p* | 177 | 19444.22 | 9.47 | nucl |
| *Aqcoe7G008000.1.p* | 218 | 24489.09 | 7.72 | chlo |
| *Aqcoe7G196400.1.p* | 210 | 23373.68 | 9.55 | nucl |
| *Aqcoe2G399900.1.p* |  |  |  |  |
| *Aqcoe6G105100.1.p* |  |  |  |  |
| *Aqcoe6G105000.1.p* |  |  |  |  |
| *Aqcoe4G235400.1.p* |  |  |  |  |
