# Supplementary material for: Comprehensive genomic analysis of the DUF4228 gene family in land plants and expression profiling of ATDUF4228 under abiotic stresses
Source: BMC Genomics. 2020 Jan 3;21:12. doi: 10.1186/s12864-019-6389-3 (PMC6942412; doi:10.1186/s12864-019-6389-3)
Supplement: Supplementary file 3 — Additional file 3: Figure S1. Details of the phylogenetic classification of DUF4228 genes in land plant lineages. Phylogenetic tree was constructed using the ML method with IQ-tree. Green lines represent bryophytes, pink lines represent pteridophytes, blue lines represent gymnosperms and red lines represent angiosperms. [file 12864_2019_6389_MOESM3_ESM.pdf]

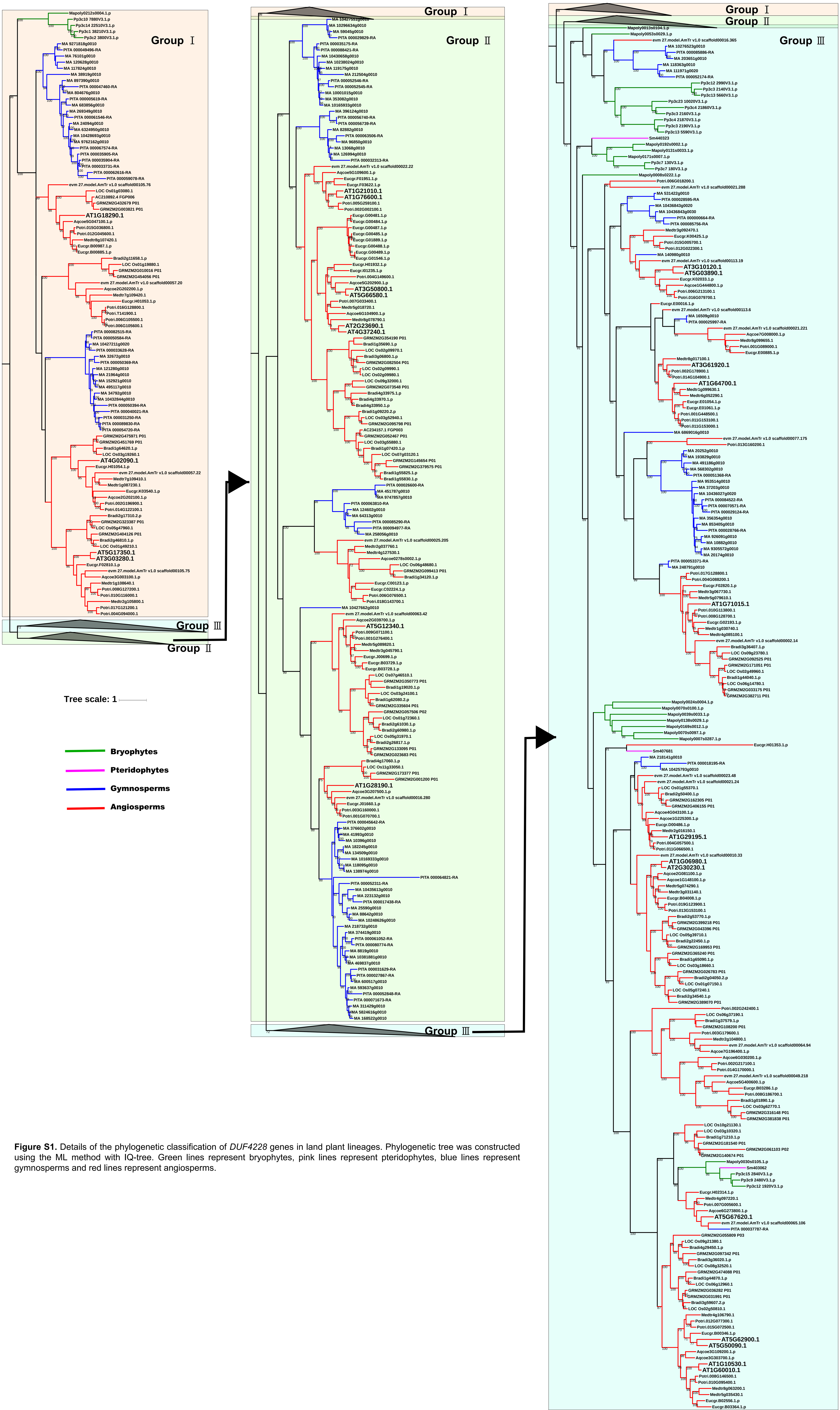

Figure S1. Details of the phylogenetic classification of *DUF4228* genes in land plant lineages. Phylogenetic tree was constructed using the ML method with IQ-tree. Green lines represent bryophytes, pink lines represent pteridophytes, blue lines represent gymnosperms and red lines represent angiosperms.
