# Supplementary figures and images for: Comprehensive genomic analysis of the DUF4228 gene family in land plants and expression profiling of ATDUF4228 under abiotic stresses
Source: BMC Genomics. 2020 Jan 3;21:12. doi: 10.1186/s12864-019-6389-3 (PMC6942412; doi:10.1186/s12864-019-6389-3)

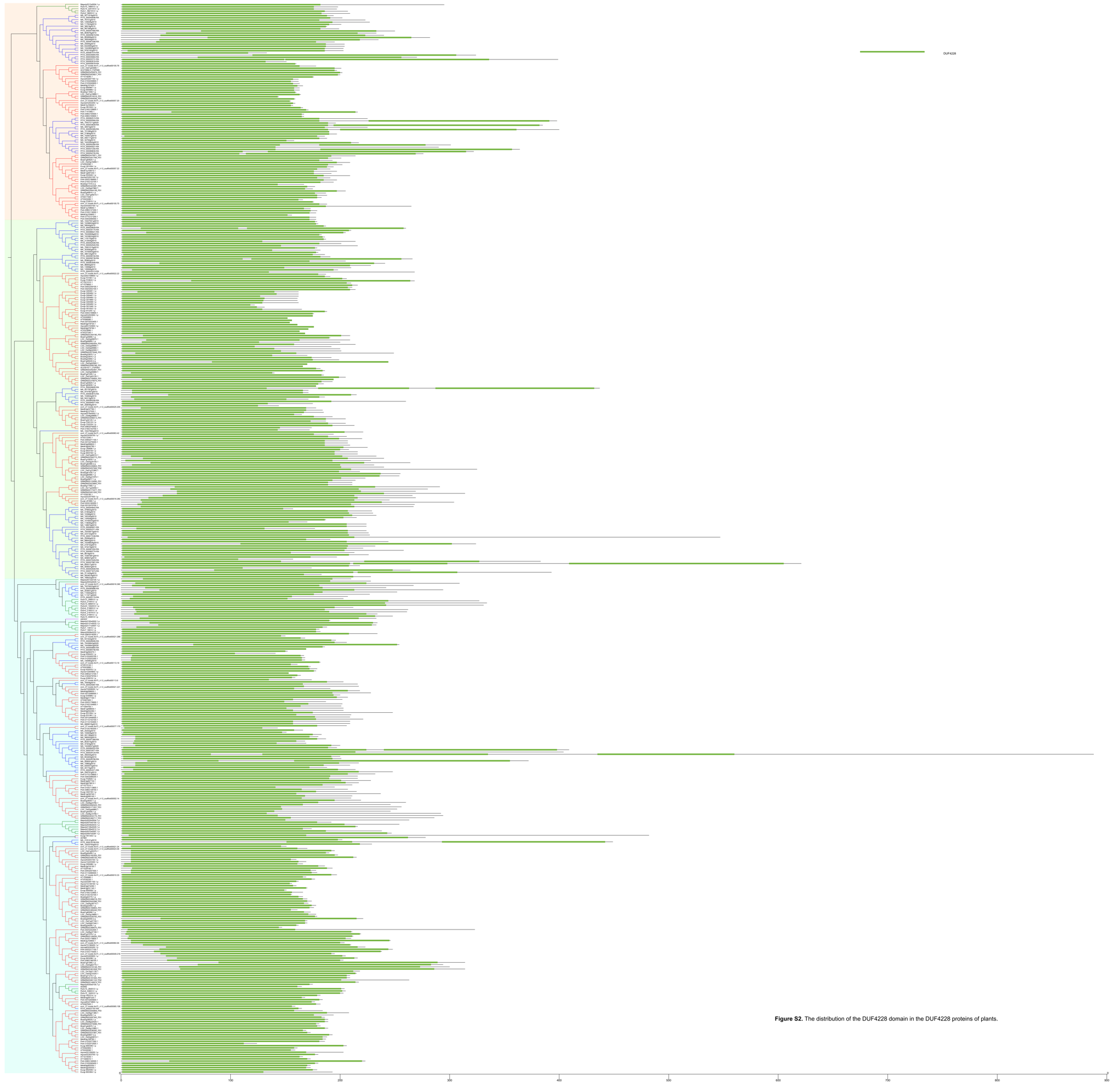

Figure S2. The distribution of the DUF4228 domain in the DUF4228 proteins of plants.

Supplement: Supplementary file 4 — Additional file 4: Figure S2. Distribution of the DUF4228 domain in the DUF4228 proteins of plants. [file 12864_2019_6389_MOESM4_ESM.pdf]

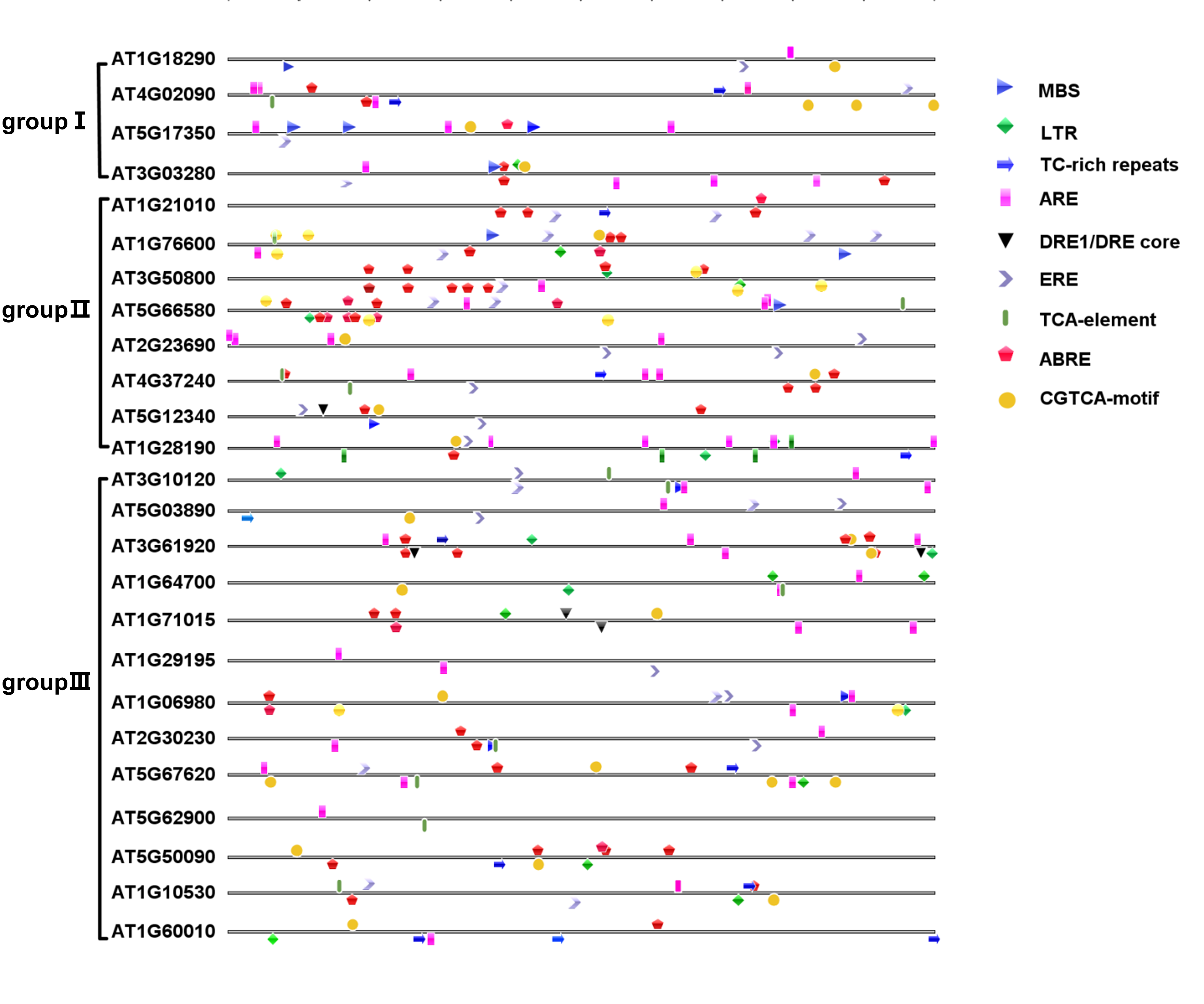

Supplement: Supplementary file 9 — Additional file 9: Figure S6. Promoter analyses of ATDUF4228 genes. The promoter sequences (2 kb upstream of ATG) of the 25 ATDUF4228 genes were analysed by PlantCARE. Cis-elements responsive to abiotic stresses and plant hormones are indicated in different colours and shapes. [file 12864_2019_6389_MOESM9_ESM.tif]

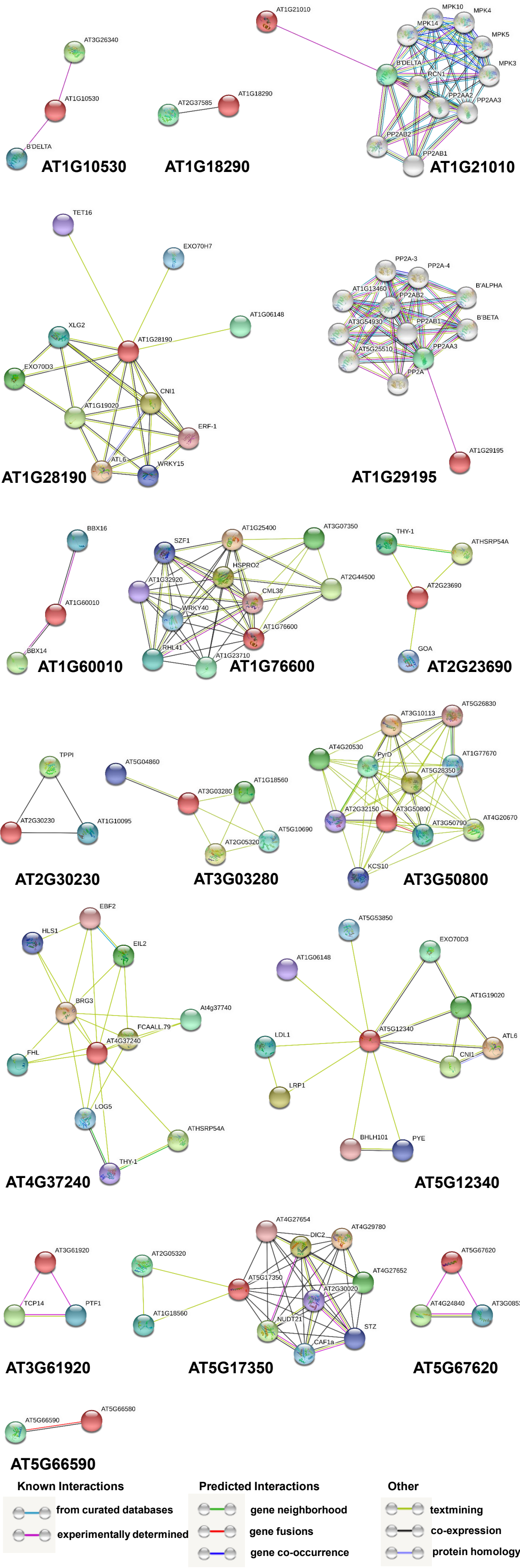

Supplement: Supplementary file 10 — Additional file 10: Figure S7. Protein-protein interaction network of ATDUF4228 proteins. In the network generated by STRINGV9.1, each node represents a protein and each edge represents an interaction, coloured by evidence type. [file 12864_2019_6389_MOESM10_ESM.pdf]
