# Supplementary material for: Comprehensive genomic analysis of the DUF4228 gene family in land plants and expression profiling of ATDUF4228 under abiotic stresses
Source: BMC Genomics. 2020 Jan 3;21:12. doi: 10.1186/s12864-019-6389-3 (PMC6942412; doi:10.1186/s12864-019-6389-3)
Supplement: Supplementary file 6 — Additional file 6: Figure S4. The distribution of ATDUF4228 genes on the chromosomes. The chromosomal position of each ATDUF4228 gene was mapped according to the genome of A. thaliana. The chromosome number is indicated at the top of each chromosome. [file 12864_2019_6389_MOESM6_ESM.pdf]

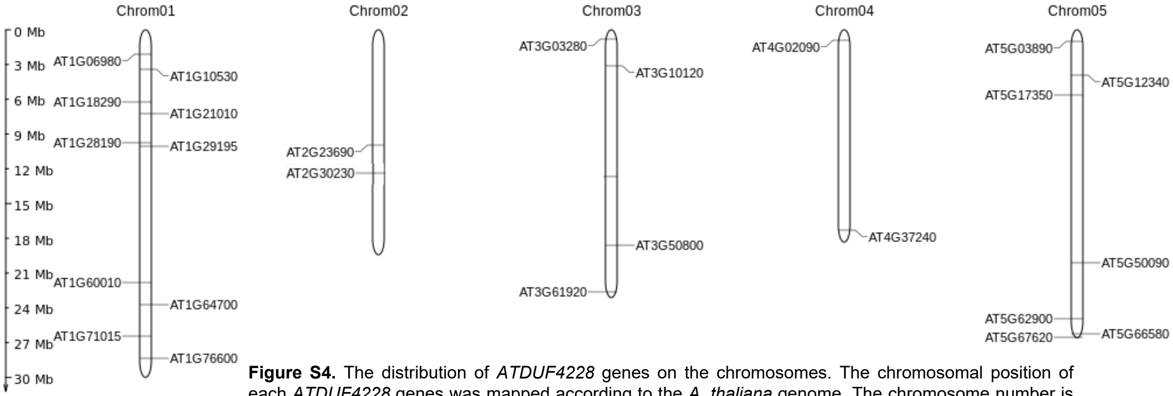

Figure S4. The distribution of *ATDUF4228* genes on the chromosomes. The chromosomal position of each *ATDUF4228* genes was mapped according to the *A. thaliana* genome. The chromosome number is indicated at the top of each chromosome.
