# Supplementary material for: Comprehensive genomic analysis of the DUF4228 gene family in land plants and expression profiling of ATDUF4228 under abiotic stresses
Source: BMC Genomics. 2020 Jan 3;21:12. doi: 10.1186/s12864-019-6389-3 (PMC6942412; doi:10.1186/s12864-019-6389-3)
Supplement: Supplementary file 7 — Additional file 7: Figure S5. Exon-intron structure of the ATDUF4228 genes. Yellow boxes: exons; blue boxes: UTRs; black lines: introns. The lengths of the boxes and lines are scaled based on gene length. [file 12864_2019_6389_MOESM7_ESM.pdf]

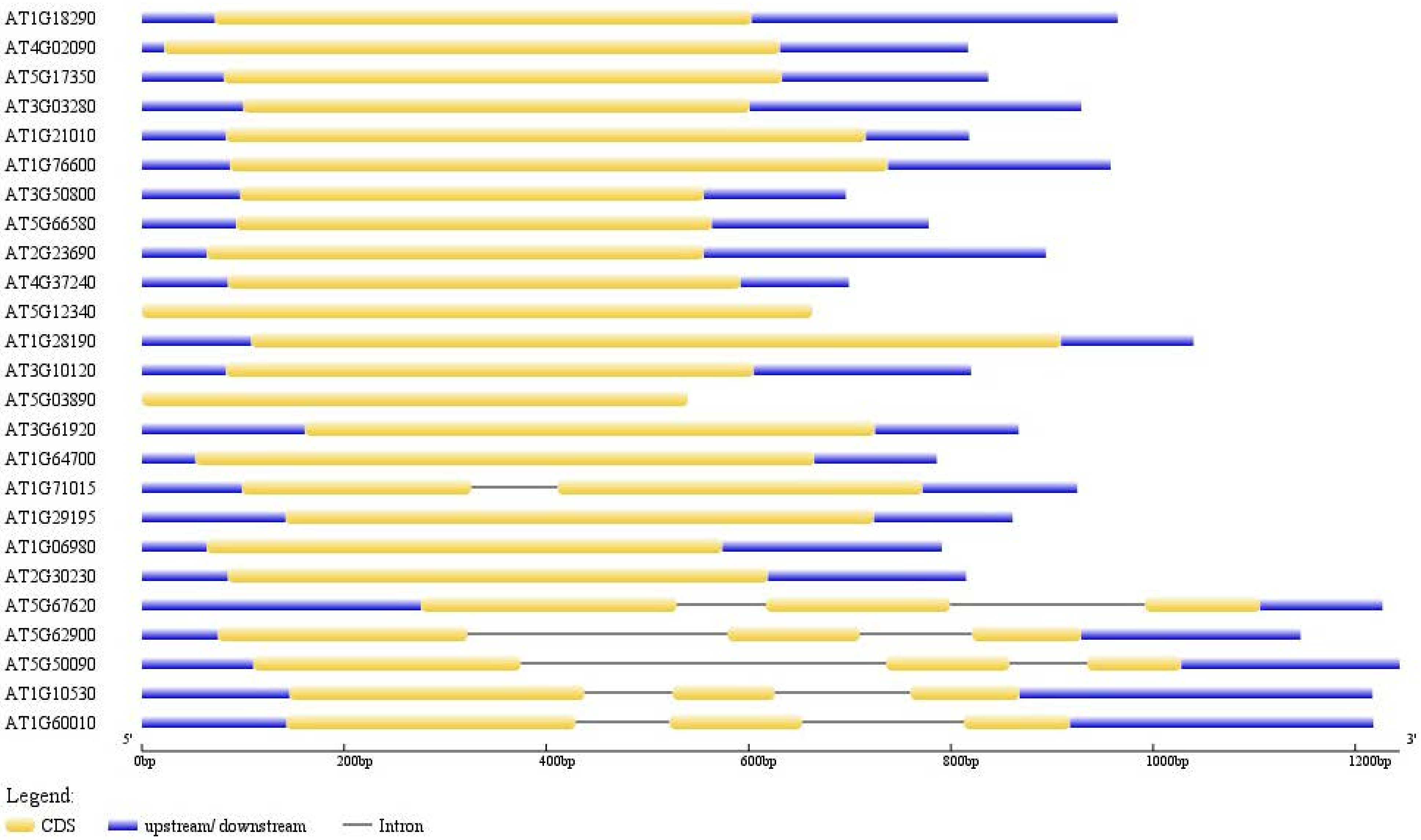

Figure S5. Exon-intron structures of the *AtDUF4228* genes. Yellow boxes: exons; blue boxes: UTRs; black lines: introns. The lengths of the boxes and lines are scaled based on gene length.
