# Supplementary material for: Comprehensive genomic analysis of the DUF4228 gene family in land plants and expression profiling of ATDUF4228 under abiotic stresses
Source: BMC Genomics. 2020 Jan 3;21:12. doi: 10.1186/s12864-019-6389-3 (PMC6942412; doi:10.1186/s12864-019-6389-3)
Supplement: Supplementary file 8 — Additional file 8: Table S3. Number and location of abiotic stress- and hormone-responsive cis-elements present in the promoter regions of ATDUF4228 genes. [file 12864_2019_6389_MOESM8_ESM.docx]

**Table S3.** Number and location of abiotic stress-responsive and hormone-responsive cis-elements present in the promoter regions of the *ATDUF4228* genes

| Group | **Gene Name** | **MBS** | **TC-rich repeats** | **C-repeat/DRE** | **LTR** | **ARE** | **ABRE** | **CGTCA-motif** | **ERE** | **TCA-element** |
| --- | --- | --- | --- | --- | --- | --- | --- | --- | --- | --- |
| groupⅠ | *AT1G18290* | 1(-174) |  |  |  | 1(-1592) |  | 1(-1717) | 1(-1459) |  |
|  | *AT4G02090* |  | 2(-474,-1392) |  |  | 4(-418,-1471,-88,-73) | 2(-392,-237) | 3(-1779,-1997,-1642) | 1(-1922) | 1(-125) |
|  | *AT5G17350* | 3(-867,-345,-188) |  |  |  | 3(-1254,-79,-624) | 1(-791) | 1(-686) | 1(-160) |  |
|  | *AT3G03280* | 1(-757) |  |  | 1(-821) | 4(-390,-1099,-1375,-1666) | 3(-780,-781,-1857) | 1(-840) | 1(-334) |  |
| groupⅡ | *AT1G21010* |  | 1(-1067) |  |  |  | 4(-772,-1509,-849,-1493) |  | 2(-925,-1379) |  |
|  | *AT1G76600* | 2(-1747,-751) |  |  | 1(-941) | 1(-85) | 4(-1053,-684,-1081,-1082) | 4(-137,-140,-228,-1051) | 4(-1832,-606,-1645,-904) | 1(-132) |
|  | *AT3G50800* |  |  |  | 2(-1073,-1449) | 1(-887) | 9(-509,-737,-1068,-634,-510,-1346,-679,-399,-400) | 3(-1325,-1679,-1443) | 1(-776) |  |
|  | *AT5G66580* | 1(-1564) |  |  | 1(-232) | 3(-1527,-676,-1519) | 9(-340,-341,-422,-932,-280,-421,-343,-165,-246) | 3(-108,-400,-1075) | 2(-754,-580) | 1(-1909) |
|  | *AT2G23690* |  |  |  |  | 4(-292,-1226,-11,-6) |  | 1(-331) | 3(-1071,-1557,-1793) |  |
|  | *AT4G37240* |  | 1(-1055) |  |  | 3(-1222,-517,-1181) | 4(-162,-1663,-1585,-1715) | 1(-1661) | 1(-694) | 2(-153,-345) |
|  | *AT5G12340* | 1(-416) |  | 1(-269) |  |  | 2(-387,-1338) | 1(-427) | 2(-212,-718) |  |
|  | *AT1G28190* |  | 1(-1919) |  | 1(-1547) | 6(-139,-745,-1544,-1997,-1181,-1418) | 1(-639) | 1(-646) | 1(-365) | 4(-1229,-326,-1492,-1595) |
| groupⅢ | *AT3G10120* | 1(-1281) |  |  | 1(-150) | 3(-1291,-1980,-1777) |  |  | 2(-820,-822) | 2(-1245,-1079) |
|  | *AT5G03890* |  | 1(-56) |  |  | 1(-1233) |  | 1(-514) | 3(-712,-1736,-1458) |  |
|  | *AT3G61920* |  | 1(-608) | 2(-1961,-527) | 1(-860) | 4(-1408,-1309,-446,-1951) | 6(-1818,-1747,-1831,-502,-649,-503) | 2(-1820,-1763) |  |  |
|  | *AT1G64700* |  |  |  | 3(-963,-1541,-1969) | 2(-1786,-1563) |  |  |  | 1(-1570) |
|  | *AT1G71015* |  |  | 2(-957,-1056) | 1(-785) | 2(-1614,-1939) | 3(-414,-475,-476) | 1(-1214) |  |  |
|  | *AT1G29195* |  |  |  |  | 2(-610,-313) |  |  | 1(-1207) |  |
|  | *AT1G06980* | 1(-1750) |  |  | 1(-1917) | 2(-1765,-1568) | 2(-118,-117) | 3(-1895,-315,-607) | 2(-1416,-1380) |  |
|  | *AT2G30230* | 1(-752) |  |  |  | 2(-303,-1680) | 2(-659,-704) |  | 1(-1495) | 1(-757) |
|  | *AT5G67620* |  | 1(-1428) |  | 1(-1628) | 3(-498,-103,-1597) | 2(-762,-1311) | 4(-1539,-1041,-121,-1719) | 1(-384) | 1(-535) |
|  | *AT5G62900* |  |  |  |  | 1(-267) |  |  |  | 1(-557) |
|  | *AT5G50090* |  | 1(-769) |  | 1(-1018) |  | 5(-1069,-1248,-296,-1068,-877) | 2(-195,-879) | 1(-1629) |  |
|  | *AT1G10530* |  | 1(-1476) |  | 1(-1444) | 1(-1274) | 2(-1468,-351) | 1(-1544) | 2(-398,-981) | 1(-315) |
|  | *AT1G60010* |  | 3(-542,-935,-1999) |  | 1(-127) | 1(-574) | 1(-1216) | 1(-353) |  |  |

The numbers in parentheses indicate the cis-elements relative position upstream of the ATG. The number that is marked in red indicate the reverse strand of DNA.
