# Supplementary material for: Comprehensive genomic analysis of the DUF4228 gene family in land plants and expression profiling of ATDUF4228 under abiotic stresses
Source: BMC Genomics. 2020 Jan 3;21:12. doi: 10.1186/s12864-019-6389-3 (PMC6942412; doi:10.1186/s12864-019-6389-3)
Supplement: Supplementary file 11 — Additional file 11: Table S4. Summary of ATDUF4228 genes with significantly altered expression (fold change≥2) after osmotic, cold and salt treatment. “up” indicates upregulated expression with a minimum 2-fold change compared with the control. “down” indicates downregulated expression with a minimum 2-fold change compared with the control. “-” indicates no obvious change detected. [file 12864_2019_6389_MOESM11_ESM.docx]

**Table S4.** Summary of *ATDUF4228* genes with significantly altered expression (fold change≥2) after osmotic, cold and salt treatment. “up” indicates upregulated expression with a minimum 2-fold change compared with the control. “down” indicates downregulated expression with a minimum 2-fold change compared with the control. “-” indicates no obvious change detected.

| Group | Gene ID | tissue | osmotic stress | cold stress | salt stress |
| --- | --- | --- | --- | --- | --- |
| Group Ⅰ | AT4G02090 | aeria | up | up | up |
|  |  | root | up | - | up |
| Group Ⅱ | AT1G21010 | aeria | up | up | - |
|  |  | root | - | - | up |
|  | AT5G66580 | aeria | down | down | down |
|  |  | root | down | up | down |
|  | AT2G23690 | aeria | down | down | down |
|  |  | root | down | down | down |
|  | AT1G28190 | aeria | up | up | up |
|  |  | root | up | down | up |
| Group Ⅲ | AT1G10530 | aeria | up | - | up |
|  |  | root | up | down | up |
|  | AT1G29195 | aeria | up | down | up |
|  |  | root | - | - | up |
|  | AT1G60010 | aeria | - | down | - |
|  |  | root | down | down | down |
|  | AT1G64700 | aeria | up | - | - |
|  |  | root | up | up | - |
|  | AT1G71015 | aeria | up | down | up |
|  |  | root | down | down | down |
|  | AT3G10210 | aeria | up | - | - |
|  |  | root | - | - | down |
|  | AT3G61920 | aeria | - | up | up then down |
|  |  | root | down | down | up then down |
|  | AT5G03890 | aeria | up | up | up |
|  |  | root | up then down | - | up |
|  | AT5G50090 | aeria | up | - | - |
|  |  | root | - | down | down |
|  | AT5G62900 | aeria | up | up | up |
|  |  | root | - | - | - |
|  | AT5G67620 | aeria | up | up | up |
|  |  | root | up | down | up |
