# Supplementary material for: Comprehensive genomic analysis of the DUF4228 gene family in land plants and expression profiling of ATDUF4228 under abiotic stresses
Source: BMC Genomics. 2020 Jan 3;21:12. doi: 10.1186/s12864-019-6389-3 (PMC6942412; doi:10.1186/s12864-019-6389-3)
Supplement: Supplementary file 12 — Additional file 12: Table S5. All primers used in this study. [file 12864_2019_6389_MOESM12_ESM.docx]

**Table S5.** All primers used in this study

| Gene | Sense/antisense-primer | Antisense-primer |
| --- | --- | --- |
| *AT1G10530* | CTAGAGTGGAGAGCCTGATCCGA | TAGACTCCATCCGTTTATCCTCTGTAAT |
| *AT1G21010* | CGGTTATGATGTTGACGGGGT | TCGTAGATGGTTGAAAGTTTTAGCC |
| *AT1G28190* | CGATAACAGAGACTAAGGCAATGAAAG | TTGGATTTGGATTGAAGTTTACGCT |
| *AT1G29195* | AACTGAAGACTGAGAAAACGGCATC | TGAATCGGAAGTTAAAATCTTTTGTTC |
| *AT1G60010* | CTGGAAACGAAAGATGAGAAGCAAC | TAAAACCCAAACATCAACTCGTAGC |
| *AT1G64700* | CCAACAGCGAGTCCTTGTCTGC | CTTTTGTCGGGTCCTTATGTGGCT |
| *AT1G71015* | GCGTGAAGTTGAAGTTACCAAAGTG | ACCTCATGTGATTTTGGCACCTT |
| *A2G23690* | GGTGATCTTTTCTGCTCGAAGAGTG | ATACTTCCTCCTCCCGCTACCAC |
| *AT3G10120* | AGCATTCCTGAAACTGATTAGCCCA | TACCAACCCTTGGAACAGTATTTCTG |
| *AT3G61920* | A ATTCAGACCAGCTAAGCGTTACGA | CTTCAATGAATACGGAAATCAACATACTAA |
| *AT4G02090* | TTTCTTAACGTGCGTATGTGTCTTG | ACGAATGAAACAGAGGTTTTGGAAT |
| *AT5G03890* | GAGGTGGAAGGCGACGAAAGATTA | CTCCAGCCGCTATTACATTCAAGAT |
| *AT5G50090* | ATTACAAAGCATATCGGAAGGAGGA | CAGCTTTTGTCATTAGCCACTCACA |
| *AT5G62900* | GCTAAAACACTTCAATCCATATTTTTCTAT | AACAATACAAAGAGAAATGAAATGATAAAA |
| *AT5G66580* | AACGACATCGGAAAAAACTTACCAA | AATGACTAAATTGACCCTACTCGGC |
| *AT5G67620* | GATCAAAAGCGTAGATATTCTTAAAGCAAC | ATTAAAGCCACAGAAAACAGTAACATCG |
