# Supplementary material for: Comprehensive genomic analysis of the DUF4228 gene family in land plants and expression profiling of ATDUF4228 under abiotic stresses
Source: BMC Genomics. 2020 Jan 3;21:12. doi: 10.1186/s12864-019-6389-3 (PMC6942412; doi:10.1186/s12864-019-6389-3)
Supplement: Supplementary file 5 — Additional file 5: Figure S3. The motif compositions of DUF4228 proteins in plants. Motifs conserved across DUF4228 proteins were identified through MEME analysis. [file 12864_2019_6389_MOESM5_ESM.pdf]

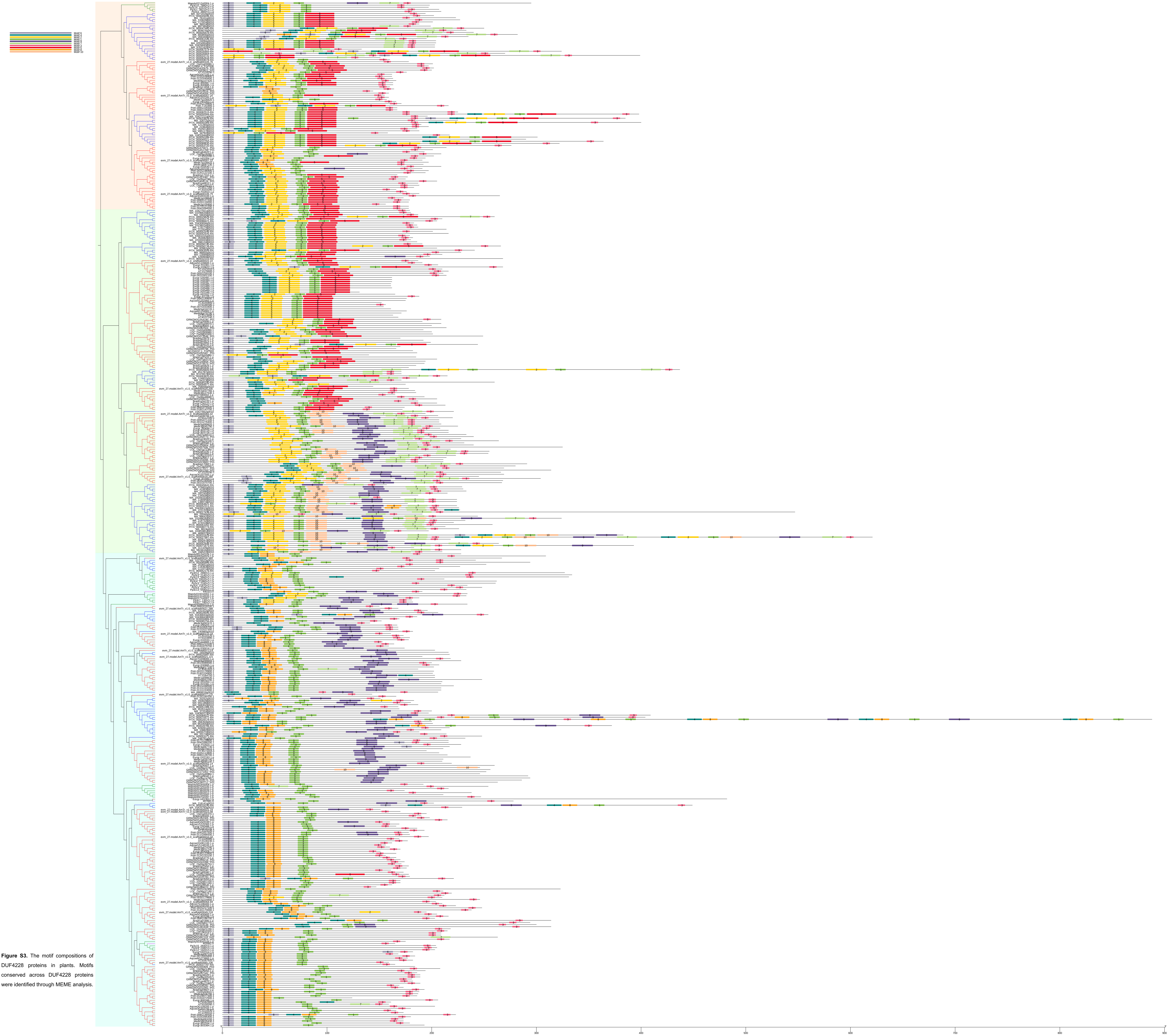

**Figure S3.** The motif compositions of DUF4228 proteins in plants. Motifs conserved across DUF4228 proteins were identified through MEME analysis.
